# Changes in net ecosystem exchange over Europe during the 2018 drought based on atmospheric observations

**DOI:** 10.1098/rstb.2019.0512

**Published:** 2020-09-07

**Authors:** R. L. Thompson, G. Broquet, C. Gerbig, T. Koch, M. Lang, G. Monteil, S. Munassar, A. Nickless, M. Scholze, M. Ramonet, U. Karstens, E. van Schaik, Z. Wu, C. Rödenbeck

**Affiliations:** 1 ATMOS, NILU – Norsk Institutt for Luftforskning, Kjeller, Norway; 2 Laboratoire des Sciences du Climat et de l'Environnement, LSCE/IPSL, CEA-CNRS-UVSQ, Université Paris-Saclay, Gif sur Yvette, France; 3 Biogeochemical Signals, Max Planck Institute for Biogeochemistry, Jena, Germany; 4 Meteorologisches Observatorium Hohenpeissenberg, Deutscher Wetterdienst, Germany; 5 Department of Physical Geography and Ecosystem Science, Lund University, Lund, Sweden; 6 School of Chemistry, University of Bristol, Bristol, UK; 7 ICOS Carbon Portal, Lund University, Sweden; 8 Meteorology and Air Quality, Wageningen University and Research, Wageningen, The Netherlands

**Keywords:** atmospheric inversion, atmospheric tracer transport modelling, net ecosystem exchange, drought

## Abstract

The 2018 drought was one of the worst European droughts of the twenty-first century in terms of its severity, extent and duration. The effects of the drought could be seen in a reduction in harvest yields in parts of Europe, as well as an unprecedented browning of vegetation in summer. Here, we quantify the effect of the drought on net ecosystem exchange (NEE) using five independent regional atmospheric inversion frameworks. Using a network of atmospheric CO_2_ mole fraction observations, we estimate NEE with at least monthly and 0.5° × 0.5° resolution for 2009–2018. We find that the annual NEE in 2018 was likely more positive (less CO_2_ uptake) in the temperate region of Europe by 0.09 ± 0.06 Pg C yr^−1^ (mean ± s.d.) compared to the mean of the last 10 years of −0.08 ± 0.17 Pg C yr^−1^, making the region close to carbon neutral in 2018. Similarly, we find a positive annual NEE anomaly for the northern region of Europe of 0.02 ± 0.02 Pg C yr^−1^ compared the 10-year mean of −0.04 ± 0.05 Pg C yr^−1^. In both regions, this was largely owing to a reduction in the summer CO_2_ uptake. The positive NEE anomalies coincided spatially and temporally with negative anomalies in soil water. These anomalies were exceptional for the 10-year period of our study.

This article is part of the theme issue ‘Impacts of the 2018 severe drought and heatwave in Europe: from site to continental scale’.

## 1. Introduction

In 2018, Europe experienced an extensive heatwave and drought. European temperatures were much higher than the 1981–2010 average from April to December, with a mean temperature anomaly of +2.5°C in May [1]. Along with the anomalously high temperatures was an extended period of low precipitation, especially over parts of Central and Northern Europe where the total precipitation in spring, summer and autumn fell below 80% of normal. In Germany, the spring and summer precipitation even fell below 40% of normal [1]. What makes 2018 stand out compared to previous heatwaves and droughts in the twenty-first century (specifically in 2003 and 2015) is its long duration, from spring to winter, which led to agricultural losses, water restrictions and, through low river levels, disruptions to shipping.

The effects of the 2003 drought on the land biosphere and net ecosystem exchange (NEE) have been widely studied [2–4]. NEE is the difference between terrestrial ecosystem respiration and gross primary production, where a positive NEE denotes a flux to the atmosphere. Ciais *et al*. [2] estimated a 30% decrease in gross primary productivity (GPP) over Europe in 2003. The decrease in GPP coincided with soil drying and water stress, which led to stomatal closure and a decrease in evapotranspiration [2]. The finding that the decline in GPP was primarily due to water, and not heat stress, is corroborated by other studies [3,4]. Terrestrial ecosystem respiration (TER) decreased coincidently with GPP, owing to reduced auto- and heterotrophic respiration, again thought to be due to the water deficit [2]. The reduction in TER did not completely compensate the reduction in GPP, and overall a reduction was also seen in NEE, and resulted in an anomalous source of CO_2_ to the atmosphere from the land biosphere in 2003 [2].

The 2018 drought also had a widespread impact on European vegetation, which could be seen from space. Both the moderate resolution imaging spectroradiometer (MODIS) on the Terra satellite and the visible infrared imaging radiometer suite (VIIRS) on the Suomi NPP satellite showed an extensive browning of vegetation in July 2018 (https://earthobservatory.nasa.gov/images/92490/heatwave-turns-europe-brown). Satellite observations of leaf area index (LAI) also showed significant anomalies, with July 2018 having the lowest LAI of any July since the start of the record in 2000, and negative LAI anomalies were seen from June to October [5]. The apparent plant stress from summer to autumn 2018 suggests that there was also an impact on NEE and carbon uptake by the land biosphere, but quantification of this impact is still needed.

In this study, we use atmospheric inversions, which assimilate observations of CO_2_ mole fractions into models of atmospheric tracer transport to constrain NEE fluxes in a Bayesian statistical framework. Atmospheric inversions have been widely used to estimate NEE on global and regional scales, and there are several examples focusing on Europe (e.g. [6–10]). Since the inversions use atmospheric CO_2_ mole fractions, they provide a constraint on NEE that is largely independent of estimates based on eddy covariance (EC) flux measurements and land surface models and thus can be considered as complementary to these approaches. However, owing to the previous lack of long-term regional observation records, and to coarse model resolution, earlier inversions provided only tenuous results on regional scales and, therefore, have not been hitherto used to assess the impact of droughts on NEE in Europe [11,12], although an inversion has been used in the study of the 2012 drought in the USA [13]. The recent inversion inter-comparison project, EUROCOM, has made significant progress on examining the robustness of European CO_2_ inversions [14], and this study applies a suite of the EUROCOM inversions to assess the impacts of the 2018 drought.

Using estimates of NEE for Europe from five different regional inversion frameworks for the past 10 years (2009–2018), we examine the impact of the 2018 heatwave and drought on NEE and compare the annual land biosphere carbon uptake to that of previous years. Moreover, we address the following questions: how well do inverse models detect inter-annual variations in NEE? And how exceptional were changes in NEE in 2018 with respect to previous years?

## 2. Methodology

### (a) Overview of the inversion method

We use five atmospheric inversion frameworks, which are all based on Bayesian statistics (table 1). Atmospheric inversions find the statistically optimal fluxes, given the observed CO_2_ mole fractions and prior statistical information about the fluxes. This is expressed as finding the fluxes (or flux parameters), **x**, that minimize the cost function:

2.1 $$\begin{array}{r} J(x)=\frac{1}{2}{\left(x-x_{b}\right)}^{T}B^{-1}(x-x_{b})+\frac{1}{2}{\left(H(x)-y\right)}^{T}R^{-1}(H(x)-y), \end{array}$$

where **x** is a vector of fluxes (or flux parameters) controlled by the inversion and **x_b_** is the prior value of **x** and **y** is a vector of observed CO_2_ mole fractions. H(**x**) is the observation operator, which relates **x** to CO_2_ mole fractions, and is described by an atmospheric transport model (see the description of the inversion frameworks in the electronic supplementary material, Information). **B** and **R** are error covariance matrices that describe the uncertainties of the prior fluxes (or flux parameters) and the uncertainties of the observations and observation operator, respectively (assuming that these uncertainties have Gaussian distributions) [17,18]. The posterior value of **x**, i.e. the solution minimizing this equation, can be found by solving the first-order derivative using a descent algorithm, also known as the variational approach, and is the approach used by four of the inversion frameworks in this study [8,9,19–21]. The fifth framework (NAME-HB) uses a Monte Carlo Markov Chain approach to find the solution for **x** [22]. The observation operator, H(**x**), represents atmospheric transport of CO_2_ and, depending on the definition of **x**, a statistical model relating the parameters **x** to NEE fluxes, where the parameters are offsets or scaling factors of first-guess estimates of NEE. The inversion frameworks included in this study are fully described in Monteil *et al.* [14] and in the electronic supplementary material, Information.

**Table 1.** Overview of the atmospheric inversion frameworks used in this study.

	CarboScope-Regional	FLEXINVERT	LUMIA	NAME-HB	PyVAR-CHIMERE
transport model	STILT (Lagrangian)	FLEXPART (Lagrangian)	FLEXPART (Lagrangian)	NAME (Lagrangian)	CHIMERE (Eulerian)
meteo. forcing	ECMWF IFS	ECMWF IFS	ECMWF EI	UK Met Office unified model	ECMWF IFS
transport resolution	0.25° × 0.25°	0.5° × 0.5°	0.5° × 0.5°	0.233° × 0.352°	0.5° × 0.5°
optimization method	variational	variational	variational	Metropolis–Hastings	variational
state vector spatial resolution	0.5° × 0.5°	variable from 0.5° × 0.5° to 4° × 4°	0.5° × 0.5°	0.35° × 0.25°	0.5° × 0.5°
state vector temporal resolution	3 h	12 h intervals averaged over 10 days	1 month	6 h	6 h
number obs. sites^a^	CSR-all: 44 CSR-select: 15 CSR-clim: 15	FI-select: 16 FI-clim: 16	LU-all: 34 LU-select: 14 LU-clim: 14	NA-select: 16 NA-clim: 16	PYV-all: 56
prior NEE	VPRM	SiBCASA	LPJ-Guess	LPJ-Guess	VPRM
prior ocean	Mikaloff-Fletcher *et al*. 2007 (fixed)	CarboScope oc_v1.6 (fixed)	CarboScope oc_v1.6 (fixed)	Takahashi *et al*. (2009) (fixed)	zero prior (optimized)
fossil fuel	EDGAR	EDGAR	EDGAR	EDGAR	EDGAR
biomass burning	none	GFEDv4.1s	none	none	none
boundary conditions	two-step approach [15]	coupling to CAMSv18r2 [16]	two-step approach [15]	coupling to CAMSv18r2 [16]	initial conditions CAMSv18r2 [16]

^a^Abbreviated names for each inversion are given, where ‘all’ indicates that all available sites were used, ‘select’ indicates that only sites with quasi-continuous measurements were used and ‘clim’ indicates that a climatological NEE prior was used but is otherwise the same as ‘select’.

### (b) Atmospheric observations

**Figure 1.**
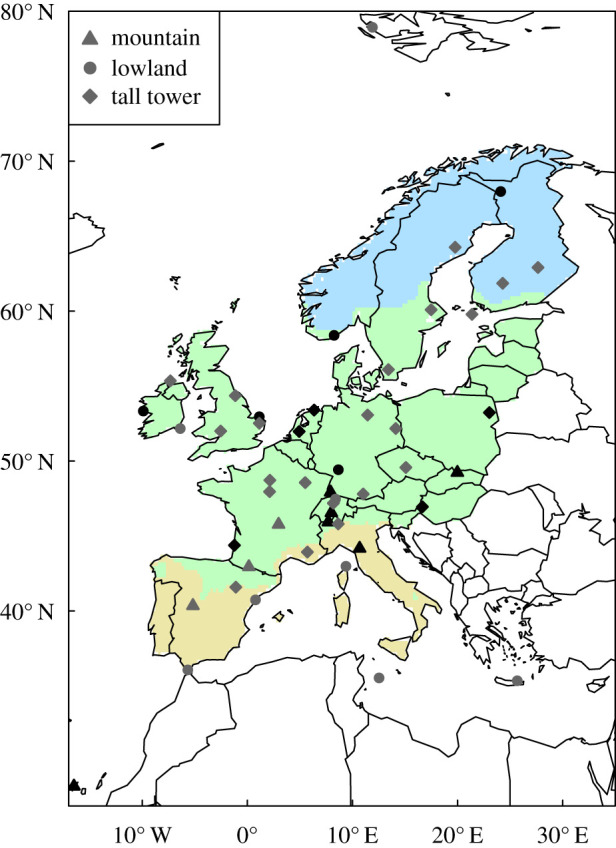
Map of observation sites. The black symbols indicate sites that have quasi-continuous observations for the period 2009–2018 (included in the ‘select’ inversions) and the grey symbols indicate all other sites. The North, Temperate and Mediterranean regions are indicated by the colour shading.

The atmospheric observations used in the inversions include *in situ* and flask measurements made at numerous sites throughout Europe (figure 1 and electronic supplementary material, table S1). Since the network is not stationary in time, as new sites were added and some sites were discontinued, we include inversions that used a selection of sites that were quasi-continuous throughout the 10-year period of our study (these are labelled ‘select’). Using a near-stationary observational dataset (i.e. one with close to the same number of observations in the same locations each year) prevents artefacts in the temporal variability of the posterior NEE that may arise due to a changing observational constraint [23]. On the other hand, selecting only quasi-continuous sites reduces the observational constraint in the inversion leading to higher uncertainties in the temporal variability of NEE. Therefore, we also include inversions using all available sites (these are labelled ‘all’), as these have a stronger constraint on NEE, especially in 2018 when more sites were operating, although changes in the observational network mean that the precision of the estimates varies from year to year.

All observation sites record mole fractions of CO_2_ in parts-per-million by volume (ppm) and are calibrated to the international standard WMO-CO2-X2007 scale. Estimates for the inter-comparability of the measurements are typically within the WMO recommended target of 0.1 ppm based on the WMO round-robin comparison experiment (https://www.esrl.noaa.gov/gmd/ccgg/wmorr/). Hourly observations were assimilated into the inversions during the daytime for low-altitude sites and during the night-time for mountain sites (for NAME-HB, a different selection criterion was used—see electronic supplementary material, Information). The selection criteria follow standard practices to avoid assimilating observations for which the model representation errors are likely to be very large, specifically for low-altitude sites at night-time and in the early morning when the planetary boundary layer is shallow, and for mountain sites when upslope winds, carrying the effect of local fluxes, may strongly influence the atmospheric signal [24,25].

Uncertainties in the observation space include estimates of the measurement error, as well as estimates of the errors from the observation operator. The quadratic sum of uncertainties for each observation gives the diagonal elements of **R** in equation (2.1) and, in all the inversions presented, the off-diagonal elements of **R** are zero, i.e. that the uncertainties are assumed to be uncorrelated. The inversion frameworks use different methods for determining observation space uncertainties, which are described in the electronic supplementary material, Information.

### (c) Prior information

Four of the atmospheric inversion frameworks only optimize NEE and thereby assume that the contribution to the model observation error from other fluxes, i.e. from the ocean, biomass burning and the combustion of fossil fuels, is negligible or at least much smaller than that from NEE. PyVAR-CHIMERE is the exception as it also optimizes ocean fluxes (but likewise assumes that the errors in the other flux components are negligible). This is the general approach used by atmospheric inversions since: (i) NEE is considered the most uncertain CO_2_ flux; (ii) on hourly time scales the atmospheric CO_2_ signal is largely dominated by NEE since the sites are located away from urban centres and thus the influence of fossil fuel emissions; and (iii) there are currently no widely available and reliable atmospheric tracers for fossil fuel emissions (e.g. ^14^CO_2_) and thus no possibility to separately constrain these in the inversions.

All inversions used gridded fossil fuel emissions based on the Emission Database for Atmospheric Research (EDGARv4.32) [26]. These data were provided at 0.1° × 0.1° and for the year 2010 and were extrapolated to other years using country-scale fuel consumption data from the BP Statistical Review of World Energy 2019. A temporally disaggregated version of the data was used with emissions estimated hourly based on sector-specific temporal factors (seasonal, weekly and diurnal) [27]. The emission of CO_2_ from fossil fuels is generally well-known for developed countries with an uncertainty typically less than the global uncertainty of 5% [28]; however, the relative uncertainty for any given grid cell or time may be much larger than that of the national total. This uncertainty is to some extent accounted for in the observation space uncertainties (see electronic supplementary material, Information), albeit imperfectly. With no widespread observations of reliable tracers for fossil fuel emissions, atmospheric inversions are still susceptible to errors in NEE due to inaccurate fossil fuel emissions [29].

For the other flux components (NEE, ocean and biomass burning), the inversion frameworks used diverse estimates (table 1). Here, we present just the prior NEE estimates used, as this component has the largest influence on the inversion results (the other components are described for each inversion in the electronic supplementary material, Information). The prior NEE models were (i) the Simple Biosphere Model - Carnegie Ames Stanford Approach (SiBCASA), which uses ECMWF ERA-Interim meteorological reanalyses to drive biophysical processes and satellite observed Normalized Difference Vegetation Index (NDVI) to track plant phenology [30]. NEE is provided at 3-hourly and 1° × 1° resolution. (ii) The Vegetation Photosynthesis and Respiration Model (VPRM), which calculates GPP based on the Enhanced Vegetation Index (EVI) from MODIS and respiration based on the temperature at 3-hourly and 0.25° × 0.25° resolution [31]. (iii) The Lund-Potsdam-Jena General Ecosystem Simulation (LPJ-GUESS) dynamic global vegetation model, which uses CRU-JRA meteorological data to estimate GPP and respiration at 6-hourly and 0.5° × 0.5° resolution [32]. Lastly, we include a set of inversions labelled ‘clim’, which use a climatological NEE prior to examine how much of the anomalies are driven by the atmospheric observations versus the prior information. Climatological fluxes of NEE were computed from each of the prior models by adjusting the 3 (or 6) hourly fluxes so that the monthly NEE of each year matched the 10-year monthly mean.

Prior uncertainties for the fluxes (or flux parameters) in **x** were used to define the diagonal elements of **B** in equation (2.1). Error correlations between variables in **x** were accounted for by including off-diagonal terms in **B**. (Details on the calculation of the prior uncertainties and the construction of **B** for each inversion framework are given in the electronic supplementary material, Information.)

### (d) Boundary conditions

For the inversion frameworks using Lagrangian transport models (FLEXINVERT, CarboScope-Regional, LUMIA and NAME-HB), the boundary conditions provide estimates of the variation in CO_2_ mole fractions that are not accounted for in the relatively short (5 to 30 days) Lagrangian model simulations. In these frameworks, the boundary condition is also known as the ‘background’ mole fraction and is described for each observation. In FLEXINVERT and NAME-HB, the background was calculated by coupling the end points of the Lagrangian backwards trajectories to 3-hourly global three-dimensional concentration fields from the CAMSv18r2 CO_2_ inversion [16] (https://atmosphere.copernicus.eu). In LUMIA and CarboScope-Regional, the background was calculated using the two-step inversion approach as described by Rödenbeck *et al*. [15] (for details, see the electronic supplementary material, Information). PyVAR-CHIMERE uses an Eulerian transport model, and in this case, the boundary condition describes the initial field of CO_2_ mole fractions for the regional model, as well as the lateral and top boundaries. The boundary conditions for this inversion were also provided by the CAMS CO_2_ inversion.

### (e) Additional datasets

In the analyses, we have used meteorological data from the ECMWF ERA5 reanalysis with 3-hourly and 30 km horizontal resolution. Specifically, the parameters used are 2 m temperature, soil water volume at 0–7 cm depth, total precipitation and downward shortwave radiation. In addition, we use the NDVI from the MODIS instrument onboard the Terra satellite. NDVI was used with 16-day and 0.05 degree resolution. For comparison with the inversion-derived NEE, we use Eddy Covariance estimates for NEE from the Fluxnet network at hourly frequency. (For access to datasets, see the acknowledgements).

## 3. Results

There is already a high level of agreement between the hourly modelled and observed CO_2_ mole fractions using the prior flux estimates with correlations at all sites better than 0.5 and model observation errors having a 1-sigma standard deviation (s.d.) of less than 8 ppm. As expected, the agreement with the observations improves further with the posterior estimates of NEE with correlations generally better than 0.7 and a s.d. of less than 6 ppm (electronic supplementary material, figures S1 and S2). The generally good agreement with the observations demonstrates a reasonable level of competence of the transport models to represent the variability of the data but does not indicate the overall performance of an inversion. The performance can to some degree be assessed by comparing with independent observations, i.e. those not included in the inversion. Although this type of validation is commonly used for inversions, it should be noted that the comparison is not strictly independent as the model observation errors for independent observations are correlated to those for assimilated observations due to the modelled atmospheric transport. Comparisons with independent observations (see electronic supplementary material, table S2) show that the agreement generally improves with the posterior NEE estimates (see electronic supplementary material, table S3 and figure S3). The one exception is LUMIA, for which there is no significant difference between the prior and posterior statistics; however, this does not indicate that this inversion framework performs more poorly than the others (and the posterior NEE is not an outlier). This is because the comparison depends on where in the domain changes to the prior NEE were made and how sensitive the modelled mole fractions are to these changes. The sites used for independent comparison include two coastal sites and three high-altitude sites, which are less sensitive to changes in NEE compared to low-altitude continental sites, but no other independent sites were available for the comparison. Furthermore, the number of and frequency of independent observations available means that this comparison is limited—the independent data are discrete samples with a frequency of 1–2 per week, thus this comparison cannot verify improvements to variability in the fluxes on the diurnal scale.

In this study, we use the results of all inversions as an ensemble of NEE estimates representing a wide range of uncertainties (i.e. random and systematic) associated with the inversion approach. The inversions give a wide range of estimates for annual NEE (electronic supplementary material, figure S4), as has been previously found [14]. This is because annual NEE is the result of a small imbalance between large CO_2_ uptake in the growing season and large CO_2_ output for the rest of the year and thus has considerable relative errors. Inversion estimates for the annual mean (2009–2018) NEE for Europe range from −0.33 to 0.37 Pg C yr^−1^, with one inversion framework (FLEXINVERT) estimating the European biosphere to be a small carbon source. FLEXINVERT finds larger winter respiration fluxes, but similar summer uptake, compared to the other inversions, resulting in a positive annual NEE. Overall though, the inversions suggest that the European biosphere is a weaker sink than the prior models indicate, with a prior range of −0.68 to −0.06 Pg C yr^−1^. Although there are large differences in the 10-year annual mean NEE among the inversions, the s.d. in annual NEE is quite consistent, showing that the inversions all show a similar degree of year-to-year variability. Moreover, all but one of the inversions (PYV-all) found more positive NEE in 2018 compared to their 10-year mean and, notably, all of the inversions using a climatological prior for NEE (these are labelled ‘clim’) find more positive NEE in 2018. This shows that the inversions are able to detect this anomaly based on the observational constraint and independently of the prior information. Furthermore, although there are differences between the ‘all’ and ‘select’ versions of a given inversion framework, the differences between frameworks are greater indicating that features specific to each framework, such as modelled transport, have a greater impact than that of changes in the observational network. This is also true for the annual and seasonal anomalies discussed below.

The 2018 drought did not affect all areas of Europe; specifically, there was a dipole with generally hotter and drier conditions than normal north of the Alps, and wetter and cooler conditions south of the Alps, that is in the Mediterranean region [33]. North of the Alps, there was also some regional variability in the onset and severity of the drought. Northern and Central Europe both experienced positive anomalies in temperature and negative anomalies in soil water from spring to autumn, whereas Western Europe was less affected in spring but had similar anomalies in summer and autumn (see electronic supplementary material, figure S5). Therefore, in the following sections, we analyse NEE for Northern and Temperate Europe (as defined by the Koeppen–Geiger Climate Classes) only, which were affected by the drought.

**Figure 2.**
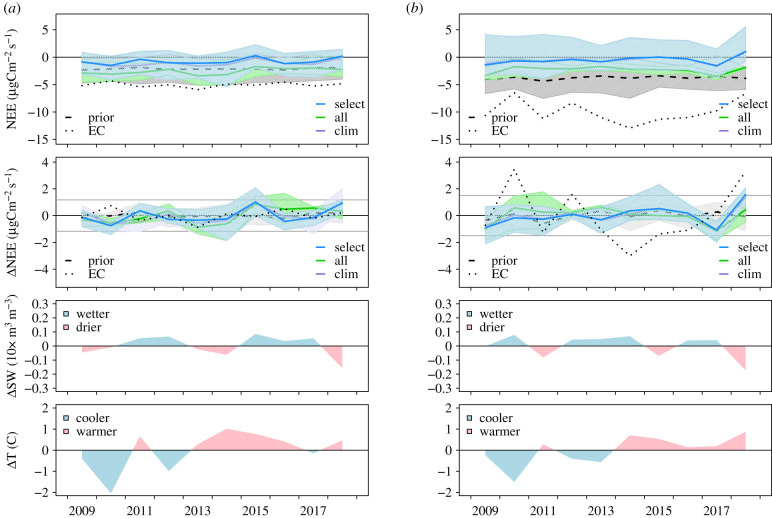
(*a*) Annual mean NEE and NEE anomaly, and annual anomalies in soil water (SW) and 2 m temperature (T) for the North region. For NEE and NEE anomaly, the means of the three inversion cases (select, all and clim) are shown by the solid lines, and the range of all inversions in each case is shown by the shading. Also shown are the mean and range of prior NEE models, VPRM, SiBCASA and LPJ-GUESS (dashed line and grey shading) and mean NEE from EC sites (dotted line). For the NEE anomaly, the horizontal grey lines show ±2 s.d. from the ensemble mean. (*b*) Similar to (*a*) but for the Temperate region.

Figure 2 shows the annual mean NEE and anomalies for the North and Temperate regions from the three sets of inversion (all, select and clim), along with the area-weighted mean anomalies in soil water content (SWC) and 2 m temperature from the ECWMF ERA5 reanalysis. In both regions, the NEE estimated by the inversions is consistently more positive (i.e. less carbon uptake) than the prior models. It is also more positive than the mean NEE from EC flux sites (see electronic supplementary material, table S4 and figure S8 for an overview of the EC sites used). Some discrepancy between the mean NEE from EC sites and the inversions is, however, expected since the EC sites have only small footprints [34] and the small number of sites covering the period of our study (17 for the Temperate and only 2 for the North region) cannot fully represent the regions as a whole.

For North Europe, the sets ‘clim’ and ‘select’ both show slightly more positive annual NEE for 2018 but still within two s.d. of the 10-year mean, while the set ‘all’ is closer to the mean, a result driven by just one inversion (CSR-all, see electronic supplementary material, figure S6). The annual NEE for 2018 was 0.02 ± 0.02 Pg C yr^−1^ (mean and s.d. of all inversions) higher than the 10-year mean of −0.04 ± 0.05 Pg C yr^−1^. By contrast, the prior models show no elevated NEE for 2018 (0.005 ± 0.017 Pg C yr^−1^ mean and s.d. of the prior models) as is also the case for NEE from the EC flux data, but with the caveat that this result is based only on two sites.

For Temperate Europe, a positive annual NEE anomaly for 2018 (i.e. at or above two s.d. of the mean) was found by the ‘select’ and ‘clim’ inversions, while the set ‘all’ also showed elevated NEE but within two s.d. owing to the result of the inversion PYV-all, which found no anomaly. On average, the annual NEE for 2018 was 0.09 ± 0.06 Pg C yr^−1^ (mean and s.d. of all inversions) higher than the 10-year mean of −0.08 ± 0.17 Pg C yr^−1^. The prior NEE models VPRM and LPJ-GUESS show only slightly more positive annual NEE for 2018 while SiBCASA shows slightly more negative NEE resulting in no mean prior anomaly (mean and s.d. of the prior models of −0.005 ± 0.069 Pg C yr^−1^). The anomaly found by the inversions is consistent with the EC flux data, which also show a distinct positive anomaly in 2018 for the Temperate region.

In both North and Temperate Europe, the reduction in CO_2_ uptake compared to the 10-year mean coincides with a decrease in soil moisture and increase in temperature, as is discussed in more detail in the next section. The results for 2018 are exceptional for the 10-year record—the only other year where the inversions show a significant anomaly is 2017 when the annual NEE was lower (i.e. greater carbon uptake) than the 10-year mean for the Temperate region.

**Figure 3.**
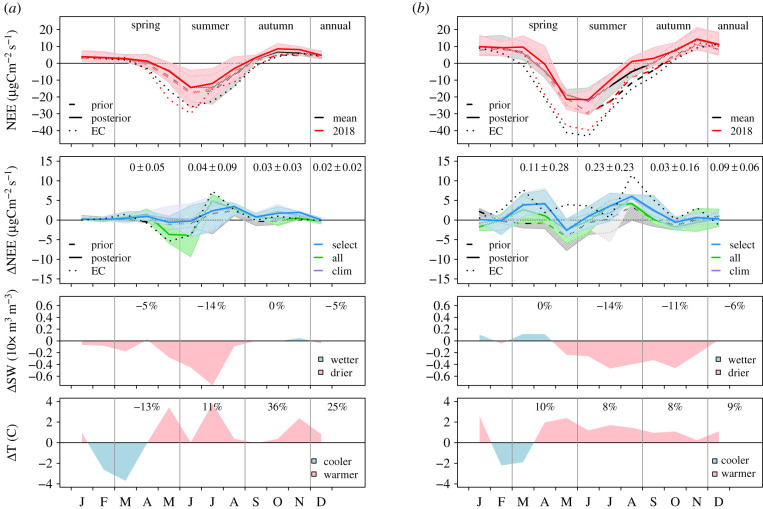
(*a*) Monthly mean NEE and NEE anomaly, and monthly anomalies in SW and T for the North region. For NEE, the mean of all months 2009–2018 is shown in black and the mean for 2018 is shown in red. The NEE anomaly is for 2018 compared to the mean 2009–2018 and is shown for the three inversion cases (select, all and clim) by the solid lines and the range of all inversions in each case is shown by the shading. The numbers give the ensemble mean and s.d. for the seasonal and annual anomalies in Pg C yr^−1^. For both NEE and NEE anomaly, the mean of prior NEE models, VPRM, SiBCASA and LPJ-GUESS (dashed line) and mean NEE from EC sites (dotted line) are also shown. For the NEE anomaly, we also show the range of the prior NEE models (grey shading). The anomalies in SW and T are for 2018 compared to 2009–2018 and the numbers give the percentage anomaly for each season and annually. (*b*) Similar to (*a*) but for the Temperate region.

Figure 3 shows the monthly mean NEE and monthly anomalies for the North and Temperate regions, along with anomalies in SWC and 2 m temperature. In the North region, the ‘all’ set of inversions shows an increase in carbon uptake for May to June, and all inversion sets show a decrease for July to August, resulting in only a small anomaly in the annual NEE of 0.02 ± 0.02 Pg C yr^−1^. The prior NEE models, in contrast, show no change in the early summer uptake, although all (but SiBCASA) show a decrease in the mid to late summer uptake, similar to the inversions. Interestingly, the monthly NEE anomalies found by the inversions are very consistent with the EC fluxes, even though these are based only on two sites. The increase in early summer uptake concurred with above-average temperatures, but the uptake decreased with a growing deficit in soil water as the summer progressed. In the Temperate region, the spring uptake is lower than average in March to April but resumed around mean levels in May, only to decrease again from June to August. This summer anomaly was found by all inversions except NA-clim. The other inversions in the ‘clim’ set, however, found a decrease in summer uptake. Of the prior models, only VPRM captured the summer decrease in carbon uptake. Similar to the North region, the reduction in summer uptake concurred with an increasing soil water deficit—14% lower than average.

**Figure 4.**
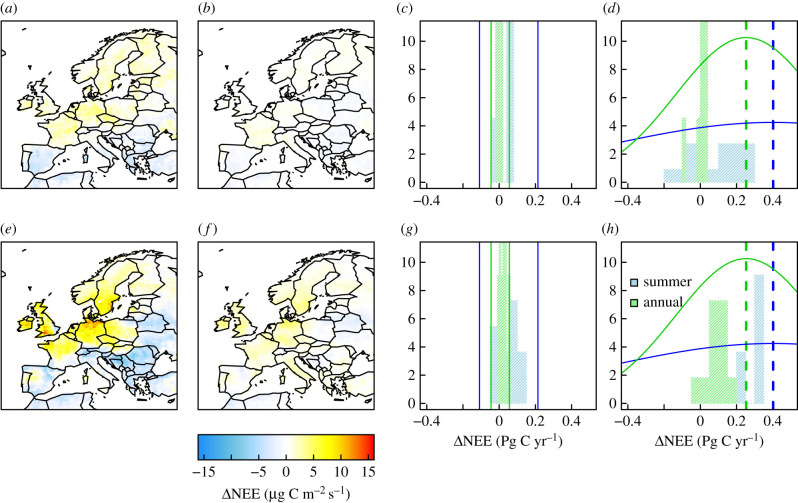
Maps of the ensemble mean NEE anomaly (*μ*g C m^−2^ s^−1^) and the inversion ensemble distributions for anomalies of the Temperate region: (*a*) prior summer anomaly, (*b*) prior annual anomaly, (*c*) prior summer and annual distributions for the North region anomaly (Pg C yr^−1^), (*d*) as for (*c*) but for the Temperate region, (*e*) posterior summer anomaly, (*f*) posterior annual anomaly, (*g*) posterior summer and annual distributions for the North region and (*h*) as for (*g*) but for the Temperate region. In (*c*) and (*g*) the anomalies for the two EC flux sites in the North region are shown (solid vertical lines). In (*d*) and (*h*), the mean summer and annual anomaly from the EC flux data (dashed lines) and the distribution given by the standard deviation across the 17 temperate EC flux sites (solid lines) are shown.

The spatial distribution of the summer and annual mean NEE anomalies for 2018 are shown in figure 4 along with histograms of the inversion ensemble results. Overall, the posterior pattern resembles that of the prior NEE models, with notable positive anomalies in Scandinavia, Germany, France, UK and Czechia but with the inversions showing stronger anomalies both for the summer and annual mean. Negative NEE anomalies in the summer are seen *a posteriori* for the Iberian Peninsula, Switzerland and southeast Europe. Note that the positive anomaly centred on northern Portugal is due solely to the inversion LU-all, which included the site Sierra de Gredos (GIC) in northwest Spain (electronic supplementary material, figure S9)—a site that appears to be influenced by very local fluxes that are not well captured by the atmospheric transport model and may result in this artefact. Notable is that the inversions show considerable convergence for the summer and annual NEE anomalies for the Temperate region, of 0.23 ± 0.25 and 0.09 ± 0.06 Pg C yr^−1^ (mean ± s.d.), respectively. The inversion ensemble is also closer to the mean summer and annual anomalies from the EC data, although there is a large spread in the anomalies across the 17 EC sites.

## 4. Discussion

The pattern of positive summer and annual NEE anomalies (figure 4) corresponds closely with positive temperature and negative precipitation and SWC anomalies (electronic supplementary material, figure S5). Furthermore, these patterns agree very closely with negative anomalies in summer NDVI from MODIS. NDVI is representative of the density and photosynthetic activity of vegetation, thus a negative NDVI anomaly indicates that the vegetation is photosynthetically less active or dense than usual.

In the North region, the increase in CO_2_ uptake (negative NEE anomaly) in May to June was correlated with positive anomalies in temperature and downward shortwave radiation. Coincidently, there was also a positive anomaly in NDVI over Norway and northern Sweden (electronic supplementary material, figure S5). Temperature is known to be a limiting factor on primary productivity in high latitudes in spring and warmer springs are generally associated with greater spring CO_2_ uptake, especially when this results in an earlier start to the growing season [35,36]. Therefore, it is likely that vegetation in Northern Europe benefited from the warmer temperatures in May leading to greater net productivity. This May to June increase in uptake, however, was offset by a decrease in the mid to late summer uptake (positive NEE anomaly), which was very likely due to a soil water deficit. The dry conditions also contributed to a particularly bad fire season in Northern Europe, particularly in Sweden, where estimates from the Global Fire Emissions Database (GFED, https://www.globalfiredata.org), suggest that the CO_2_ emitted from wildfire was approximately 10 times that of the previous year. Since NEE represents the balance of GPP and TER it excludes CO_2_ emitted through disturbances. The inversions, however, calculate NEE as the net land biosphere to atmosphere flux and rely on prior estimates of wildfire emissions to correct this flux to NEE. This correction likely did not contribute to any significant error in our NEE estimates as the CO_2_ emitted from fires in the North region in 2018 from GFED amount to 1.5 × 10^−4^ Pg C yr^−1^, which is only 0.3% of the summer NEE anomaly.

For the Temperate region, there was no significant change in the early summer (May) carbon uptake, possibly because, when temperatures began to increase in April, there was already below average SWC. In regions already with a spring deficit in soil water, such as in Germany, there was also lower than average NDVI and a positive NEE anomaly (electronic supplementary material, figures S5 and S9). By summer, a negative NDVI anomaly was apparent for the whole Temperate region and the annual anomaly was below 1 s.d. from the 10-year mean. The only other year with a comparable anomaly was 2010. The anomaly in SWC for the Temperate region exceeded 2 s.d. from the 10-year mean and was the only year for which this was the case.

Buras *et al*. [33] examined anomalies in the climate water balance (CWB), that is the difference between precipitation and potential evapotranspiration, and found large negative CWB anomalies over Northern and Central Europe during the 2018 drought, which were co-located with large negative NDVI anomalies. Buras *et al*. [33] suggest that the strong coupling of soil water deficit and NDVI may be because temperate forests are poorly adapted to dry conditions compared to those of typically more arid regions.

Overall the inversions found the European land biosphere (i.e. all three regions, 3.98 × 10^6^ km^2^) to be close to carbon neutral in 2018 with mean and median of 0.01 and −0.08 Pg C yr^−1^, respectively, and range of −0.24–0.54 Pg C yr^−1^ compared to the 10-year mean, median and range of −0.11, −0.22 and −0.33–0.37 Pg C yr^−1^. This is in contrast with the 2003 drought during which the European land biosphere (4.6 × 10^6^ km^2^) was estimated to be a source of carbon of 0.5 Pg C yr^−1^ [2]. This is even though the 2018 drought had a larger extent (24–38 Mha) compared to the 2003 drought (20–28 Mha) [37]. The greater impact on NEE in 2003 may be due to the very dry spring (from February) exacerbating the drought, while in 2018, a widespread soil water deficit is only apparent from April. In 2003, the reduction in carbon uptake was found to be due to a limitation of water, and not to extreme heat [3], which, given the strong temporal and spatial correlations of the NEE and SWC anomalies, appears to be the case also for 2018. This is also consistent with conclusions drawn from global vegetation models investigating the drought [37].

Persistent high-pressure systems, like that in 2018, may become more common in the future, particularly during the summer, due to a weakening mid-latitude circulation, which likely will lead to a greater occurrence of weather extremes and drought [38,39]. Previous studies have shown that warmer temperatures alone have a positive effect on net productivity in Northern Europe through an increase in the remineralization of organic matter and thus an increase in the supply of nitrogen, and if the warming occurs in spring, through a longer growing season [40,41]. However, in areas with reduced precipitation, the potential increase in productivity through warmer temperatures could be reduced or even offset due to deficits in soil water [40]. The inversion results for the North region in 2018 further suggest that a warmer spring can lead not only to enhanced net productivity but also to NEE, but that this enhancement can be offset by reduced NEE in summer owing to a soil water deficit. In the Temperate region, by contrast, there was no net increase in the spring uptake and the summer uptake was reduced owing to a soil water deficit.

## 5. Conclusion

The European drought of 2018 was exceptional for the last 10 years in terms of its severity and extent, as seen in the meteorological anomalies of temperature, precipitation and soil water volume, as well as in its effect on NDVI. The inversion ensemble showed a positive annual NEE anomaly (less CO_2_ uptake) in the Temperate region of 0.09 ± 0.06 (mean ± SD) Pg C yr^−1^, compared to the 10-year mean of −0.08 ± 0.17 Pg C yr^−1^, making the region close to carbon neutral for 2018. The anomaly was largely owed to reduced summer uptake with NEE being 0.23 ± 0.25 Pg C yr^−1^ more positive than average. In the North region, the annual anomaly was smaller, 0.02 ± 0.02 Pg C yr^−1^, because a negative anomaly (increased CO_2_ uptake) in May to June partially offset a summer positive anomaly. In both the North and Temperate regions, the summer decrease in CO_2_ uptake was likely driven by a soil water deficit and was visible in a reduction in NDVI.

Overall, the atmospheric inversion approach was able to detect large changes in NEE. However, the absolute value of NEE from inversions remains very uncertain. Improvements in inversion estimates could be achieved by more reliable spatiotemporally resolved estimates of fossil fuel emissions, constraints on boundary conditions and improved atmospheric transport modelling, but also through the long-term maintenance of high-density atmospheric observation networks.

## Supplementary Material

Supplementary Information for ‘Changes in net ecosystem exchange over Europe during the 2018 drought based on atmospheric observations’

## Data accessibility

The observational data and inversion results are available from the ICOS Carbon Portal: Drought 2018 Team and ICOS Atmosphere Thematic Centre: Drought-2018 atmospheric CO_2_ Mole Fraction product for 48 stations (96 sample heights), (doi:10.18160/ERE9-9D85), 2020. Drought 2018 Team and ICOS Ecosystem Thematic Centre: Drought-2018 ecosystem eddy covariance flux product for 52 stations in FLUXNET-Archive format, (doi:10.18160/YVR0-4898), 2020. Thompson, R., Broquet, G., Gerbig, C., Monteil, G., Lang, M. and Koch, F.-T.: Drought-2018 ensemble of inversion results for 2009–2018, (doi:10.18160/YQ8P-P7CF), 2019.
