# Supplementary material for: Changes in net ecosystem exchange over Europe during the 2018 drought based on atmospheric observations
Source: Philos Trans R Soc Lond B Biol Sci. 2020 Sep 7;375(1810):20190512. doi: 10.1098/rstb.2019.0512 (PMC7485096; doi:10.1098/rstb.2019.0512)

Supplementary Information

Changes in Net Ecosystem Exchange over Europe During the 2018 Drought Based on Atmospheric Observations

R. L. Thompson[1], G. Broquet[2], C. Gerbig[3], T. Koch[3,4], M. Lang[2*], G. Monteil[5], S. Munassar[3], A. Nickless[6], M. Scholze[5], M. Ramonet[2], U. Karstens[7], E. van Schaik[8], Z. Wu[5] and C. Rödenbeck[3]

1. NILU – Norsk Institutt for Luftforskning, Kjeller, Norway
2. Laboratoire des Sciences du Climat et de l'Environnement, LSCE/IPSL, CEA-CNRS-UVSQ, Université Paris-Saclay, Gif sur Yvette, France
3. Max Planck Institute for Biogeochemistry, Jena, Germany
4. Deutscher Wetterdienst, Germany
5. Dep. of Physical Geography and Ecosystem Science, Lund University, Lund, Sweden
6. School of Chemistry, University of Bristol, Bristol, UK
7. ICOS Carbon Portal, Lund University, Sweden
8. Wageningen University and Research, Wageningen, The Netherlands

*Now at Department of Meteorology, University of Reading, Reading, Berkshire, UK

Descriptions of the inversion frameworks

1. FLEXINVERT

FLEXINVERT is a Bayesian inversion framework utilising the FLEXPART Lagrangian particle dispersion model [1,2]. FLEXPART was used in a backwards time mode to model the relationship between observed CO_2 mole fractions and surface fluxes (the so-called source-receptor relationship, SRR, or "footprints") following the method of Seibert and Frank [3]. Simulations of virtual particles were made using ECMWF IFS wind fields for every hourly observation and were traced for 5 days backwards in time. The SRRs were saved globally at 0.5°×0.5° and hourly resolution giving 120 fields for each observation. These were used to describe the atmospheric transport function H(**x**) in Eq. 1. To account for the influence of atmospheric transport and fluxes prior to the end of the 5-day simulations, a so-called background mole fraction was calculated for each observation. The background consisted of two parts: 1) the influence of CO_2 mole fractions where the virtual particles terminated, which was calculated as a weighted average of 3D fields of optimized initial mole fractions from the CAMSv18r2 CO_2 inversion [4]; and 2) the influence of CO_2 fluxes along the backwards trajectories outside the inversion domain, which was calculated as the integral of the SRRs and prior fluxes outside the domain (see Ref [5]).

FLEXINVERT optimized a state vector consisting of offsets to the prior NEE fluxes on an irregular spatial grid with a resolution of between 0.5°×0.5° and 4.0°×4.0° depending on the SRRs (see Ref [5]). The offsets were optimized for 12-hour periods (00:00-12:00 and 12:00-00:00 local time) averaged over 10-days. Prior uncertainties were calculated for each offset as 25% of the absolute value of the 12-hour mean flux in each grid cell. These were used to calculate the diagonal elements of the prior error covariance matrix (**B** in Eq. 1) and the off-diagonal elements were calculated using an exponential decay model with a spatial correlation length of 200 km over land and 1000 km over ocean and 10 days in time. The prior error covariance matrix was scaled to give a total uncertainty of 0.3 PgC y^{-1} over the inversion domain. Only NEE was optimized, and the influence of fossil fuels, biomass burning, and ocean fluxes were modelled using prior flux estimates and the SRRs. Ocean fluxes were from the observation-based estimate of Rödenbeck et al. [6] using surface ocean pCO_2 measurements

and were at 5°×4° and monthly resolution. Biomass burning emissions were from the Global Fire Emissions Database (GFED-v4.1s) and were at 0.25°×0.25° and monthly resolution [7].

Observations were assimilated at hourly frequency between 12:00-16:00 local time for low altitude sites (<1000 masl) and 00-04:00 for mountain sites. A consistent dataset of 16 sites was used for each year giving approximately 24000 observations annually. Observation-space uncertainties were calculated as the quadratic sum of uncertainties for the measurement, the initial mole fractions, and the uncertainty of the fossil fuel emissions projected into the observation-space. For the latter component, the fossil fuel emissions were assumed to have an uncertainty of 25%. For the initial mole fraction uncertainty, a fixed value of 1.0 ppm was used based on the mean model-observation error at background sites. Total observation uncertainties typically varied from 1.0 to 3.0 ppb. The observation error covariance matrix (**R** in Eq. 1) was diagonal.

The optimal state vector was found using the M1QN3 Quasi-Newton algorithm.

2. LUMIA

LUMIA is a regional atmospheric inversion system developed at Lund University [8]. The current set-up is similar to the one used in Monteil et al. [9]. NEE is optimized monthly at grid scale (for the standard European domain at a horizontal resolution of 0.5°×0.5°) using a variational inversion approach.

The transport (forward and adjoint) of fluxes within the domain is calculated using footprints from the FLEXPART Lagrangian transport model [1,2]. Footprints were calculated from FLEXPART runs of 7 days backwards in time from each observation. The impact of background and historical CO_2 fluxes on the observations is computed using the 2-step approach of Rödenbeck et al. [10]. In this approach, the global (coarse resolution) CO_2 inversion is performed with the TM5-4DVAR model [11]. FLEXPART and TM5 are both driven by ECMWF ERA-Interim reanalyses.

Uncertainties on the prior NEE are set to 50% of the monthly NEE, but with a minimum value of 1% of the largest uncertainty (to avoid having too small uncertainties where NEE is close to zero because GPP and TER compensate each other). The ocean flux is based on the pCO_2 inversion of Rödenbeck et al. [6] and is not further optimized.

Observations at each site are selected based on the "dataset_time_window_utc" flag in the metadata of the observation files from the EUROCOM project protocol [described in 9]. That corresponds, for low elevation sites, to a 11:00 to 15:00 UTC time range, and to a 23:00 to 03:00 UTC time range for mountain sites. At sites with only flask observations, all samples were used. The observation uncertainties are defined as the quadratic sum of the measurement uncertainties, of the uncertainty of the foreground transport model (i.e. FLEXPART) and of the uncertainty on the background mole fractions. The measurement uncertainties are taken from the data files when available, and a minimum uncertainty of 0.3 ppm is enforced. Foreground transport model uncertainties are computed by performing two similar forward model runs, with TM5 and LUMIA (TM5+FLEXPART), configured such that the only difference is the model used to compute the transport within the EUROCOM domain. The uncertainties on the background mole fractions are set as the standard deviation of the vertical profile of background CO_2 mole fractions around each observation (see Ref [8] for details about the approach). The combined observation-space uncertainty is typically in the order of 4 ppm.

3. CarboScope-Regional

CarboScope-Regional is the regional version of the Bayesian inversion framework, CarboScope [12] using the STILT Lagrangian particle dispersion model [13] in backwards time

mode. Simulations of particle ensembles were made using ECMWF IFS short-term forecasted meteorological fields for every hourly observation and were traced for 10 days backwards in time. The surface layer (where the observations are sensitive to the fluxes) is defined in STILT as half the height of the planetary boundary layer, at any given time. The SRRs were saved for the domain of interest (35°-73°N, 15°W-35°E) at 0.25°×0.25° and hourly resolution, describing the atmospheric transport function H(**x**) in Eq. 1. The background concentrations are computed directly at each observation site by a global, coarse resolution CarboScope CO_2 inversion [12], following the 2-step approach proposed by Rödenbeck et al. [10].

CarboScope-Regional optimized a state vector consisting of offsets to the prior NEE fluxes on a regular spatial grid with a resolution of 0.5°×0.5°. Prior uncertainties and their temporal and spatial correlation scales were set following Kountouris et al. [14] (inversion "nBVH"), using a hyperbolic spatial correlation function with a length scale of 100 km (at which the correlation drops to 1/e). This resulted in a domain-integrated annual total uncertainty of 0.3 PgC y^{-1}. Only NEE was optimized, and the influence of fossil fuel emissions and ocean fluxes were modelled using prior flux estimates and the SRRs. The fossil fuel flux estimates were hourly. The ocean prior is based on the optimized data product from Mikaloff-Fletcher [15], which is a climatology with monthly and 4°×5° resolution. No prior was used for biomass burning.

Observations were assimilated at hourly frequency between 11:00-16:00 UTC for low altitude sites (<1000 masl) and 23:00-04:00 UTC for mountain sites. Two inversions were included, which use differing numbers of sites: 1) CSR-select: using a consistent dataset of 15 sites with quasi-continuous coverage throughout the 2009-2018 period, and 2) CSR-all: using 46 sites giving maximum spatial coverage. Observation-space uncertainties were calculated as follows: a base representation error of 1.5 ppm was assumed for tall towers, coastal and mountain sites. For ground based continental sites, it was raised to 2.0 ppm, and to 4 ppm for sites in urban environments (applied only for Heidelberg). For sites that provide hourly observations, an error inflation was applied (e.g. for tall towers: 1.5 ppm $\times\sqrt{6}$ obs/day × 7 day/week = 9.7 ppm). The observation error covariance matrix (**R** in Eq. 1) was diagonal.

4. NAME-HB

The NAME-HB framework uses the Lagrangian particle dispersion model, NAME. This model provides "footprints" for each observation (see [16]). In NAME-HB, 2-hourly observation time steps were used. To represent each observation, 20000 particles were released, and traced backwards in time for 30 days, so that by the end of this period the majority of particles would have left the inversion domain. The instances where the particles are in the lowest 40m of the atmosphere were recorded and this was used to represent the sensitivity of observed mole fractions to surface fluxes in the inversion domain. The domain used to calculate atmospheric transport was originally set to constrain emissions from the United Kingdom. It covers most of Europe, the east coast of North and Central America, and North Africa (10.729–79.057° N and 97.9°W–39.38°E). The meteorological analysis dataset used to drive the model is from the Met Office Unified Model, with a spatial resolution of 0.233°×0.352° (approximately 25 km by 25 km over the UK).

To account for diurnal variation in CO_2 fluxes, changes in surface sensitivity on timescales shorter than the duration of the simulation were captured (for details see [17]). This process involved obtaining the footprints for 2-hourly averaged periods backwards in time for the first 24 hours before an observation. These time-disaggregated footprints were used to replace the first 24 hours of integrated sensitivities. Mole fraction changes due to land biosphere fluxes were simulated by multiplying these footprints by biospheric flux estimates for the corresponding time. The contribution to changes in mole fractions from the fixed flux components (i.e. ocean and fossil fuel) were similarly calculated by multiplying the footprints

with prior estimates of these fluxes. The ocean prior was based on the climatology of Takahashi et al. [18]. The original spatial resolution was 4°×5° (latitude by longitude), and this was interpolated to a monthly concentration at a spatial resolution of 0.233°×0.352°.

The NAME-HB inversion assimilated 2-hourly observations with a data selection criterion based on the localness and modelled lapse rate at each site, which is a measure of atmospheric stability. The localness of observations was based on the ratio of the NAME footprint magnitude in the 25 grid cells in the immediate vicinity of the measurement site to the total for all grid cells in the domain. A high ratio indicates times when a significant fraction of air influencing the observation originates from very local sources, which may not be resolved by the model. The lapse rate is calculated as the slope of potential temperature with altitude. A high lapse rate suggests very stable conditions, which would again suggest significant local influence. The threshold for the lapse rate was selected somewhat subjectively to preserve as much data as possible, whilst retaining only points that the model was found to resolve well. Following the methodology used in White et al. [17] the localness threshold and lapse rate threshold were normalised to 500 m and a threshold value of 1.3 for the sum of these metrics was used.

The uncertainty in the observation space was composed of measurement and model uncertainty components. The measurement uncertainty was assumed to be equal to the standard deviation of the measurements over the 2-hour period to give an estimate of measurement repeatability and a measure of the sub-model-timescale variability in the observations. The 2-hourly measurement uncertainty was then averaged over the month to ensure that measurements of high concentrations were not given less weight, as they are more likely to have greater variability and therefore a larger standard deviation. The monthly average measurement uncertainty was ~0.9 ppm. The measurement uncertainty was combined with a range of prior values for model uncertainty and together the observation space uncertainty is one of the hyper-parameters optimized using a hierarchal Bayesian MCMC framework [19]. In the hierarchical Bayesian framework , "hyperparameters" that define the prior flux and model–data "mismatch" uncertainty PDFs are included in the optimization, which is performed using the Metropolis–Hastings Markov Chain Monte Carlo algorithm. A hyper-parameter was included that describes the size of the inversion grid with respect to time, following the trans-dimensional inversion approach described in Lunt et al. [20] and White et al. [17].

The domain of NAME was split into eight boxes: four "background" boxes outside the inversion domain, and four "foreground" boxes within the inversion domain. The latter were further divided based on a Plant Functional Type (PFT) map (with 6 PFTs) from the JULES vegetation model [21]. The inversion optimizes separately the gross primary production (GPP) and the heterotrophic respiration (TER) (with NEE=GPP+TER). The prior estimates of GPP and TER over Europe were obtained from LPJ-GUESS [22]. Estimates from terrestrial regions outside of Europe were taken from a 2015 simulation of the ORCHIDEE model [23]. In the hierarchical Bayesian framework, parameters defining the distribution of scaling parameters for GPP and TER were optimized within each of the PFT regions. The prior mean value for the scaling parameter was set at 1 for each PFT, with a standard deviation of 1. Both the mean and standard deviation were optimized in the inversion. The flux components are optimized at a variable temporal resolution, with a maximum resolution of one day (for details see [17]).

The prior estimates of the background concentrations were provided by the CAMS LMDZ (CAMSv18r2) CO_2 inversions [4]. The original spatial resolution of the CAMS product was 3.75°×1.85° with 39 vertical levels. This product was regridded to a spatial resolution of 0.233°×0.352° with 20 vertical levels up to an altitude of 19 500 masl. Sensitivity of observations to these 3D concentration fields was determined using the time and location when particles in the NAME model exited the domain. The mole fractions at each domain edge (N,

E, S, W) were scaled up or down during the inversion to account for uncertainties in the CAMS boundary conditions.

5. PyVAR-CHIMERE

PyVAR-CHIMERE is a regional atmospheric inversion system developed at the Laboratoire des Sciences du Climat et l'Environnement (LSCE) [Broquet:2011et; 24]. It is based on the regional Eulerian atmospheric chemistry-transport model CHIMERE [25], on its adjoint code, and on a variational data assimilation platform [26]. In this study, the PyVAR-CHIMERE inversions are set-up in a manner that is very close to that of Broquet et al. [27].

A European configuration of CHIMERE is used; this configuration covers latitudes 31.5-74°N and longitudes 15.5°W -35°E with a 0.5°×0.5° horizontal resolution and 29 vertical layers up to 300 hPa. Meteorological forcing for CHIMERE is generated using the European Centre for Medium Range Weather Forecasting (ECMWF) operational forecasts. Initial, lateral and top boundary conditions for CO_2 concentrations are generated from the CAMS global CO_2 inversions (v16r1 for 2009-2015 and v18r2 for 2016-2018) [4].

The inversion assimilates 1-hour averages of the measured CO_2 mole fractions during the time windows 12:00-18:00 UTC for low altitude stations (below 1000 masl) and 0:00-6:00 UTC for high altitude stations (above 1000 masl). The inversion optimizes 6-hourly mean NEE and ocean fluxes at the 0.5°×0.5° resolution of CHIMERE. The prior estimate of NEE and its uncertainty covariance matrix are specified using the VPRM model simulations of NEE and respiration, respectively, following the general approach of Broquet et al. [28]. The temporal and spatial correlation scales for the prior uncertainty in NEE are set to ~1 month and 200 km (following the diagnostics of Kountouris et al. [29], with no correlation between the four 6-hour windows of the same day. The ocean prior is simply a flux of zero with uncertainties that have monthly temporal and 1000 km spatial correlations. No prior is used for biomass burning.

The observation error covariance matrix (**R** in Eq. 1) is set-up to be diagonal, ignoring the correlations between errors for different hourly averages of the CO_2 measurements (which has been justified by the analysis of Broquet et al. [28]. The variances for hourly data are based on the values from Broquet et al. [27], which vary depending on the sites and season, and which are derived from Radon model-data comparisons [28].

The cost function associated with the variational scheme is minimised using the M1QN3 Quasi-Newton algorithm. The adjoint of CHIMERE is used to compute the gradient of the cost function at each iteration. To cover the whole analysis period (2009-2018), a series of 1-year inversions is performed. Posterior estimates of NEE at 1-hourly and 0.5°×0.5° resolution were generated for the full period of analysis.

References

1. Stohl, A., Forster, C., Frank, A., Seibert, P. & Wotawa, G. 2005 Technical note: The Lagrangian particle dispersion model FLEXPART version 6.2. *Atmos. Chem. Phys.* **5**, 2461–2474. (doi:10.5194/acp-5-2461-2005)

2. Pisso, I. et al. 2019 The Lagrangian particle dispersion model FLEXPART version 10.3. *Geosci. Model Dev.* **2019**, 1–67. (doi:10.5194/gmd-2018-333)

3. Seibert, P. & Frank, A. 2004 Source-receptor matrix calculation with a Lagrangian particle dispersion model in backward mode. *Atmos. Chem. Phys.* **4**, 51–63. (doi:10.5194/acp-4-51-2004)

4. Chevallier, F. et al. 2010 CO_2 surface fluxes at grid point scale estimated from a global 21 year reanalysis of atmospheric measurements. *J. Geophys. Res.* **115**. (doi:10.1029/2010JD013887)

5. Thompson, R. L. & Stohl, A. 2014 FLEXINVERT: an atmospheric Bayesian inversion framework for determining surface fluxes of trace species using an optimized grid. *Geosci. Model Dev.* **7**, 2223–2242. (doi:10.5194/gmd-7-2223-2014)

6. Rödenbeck, C., Keeling, R. F., Bakker, D. C. E., Metzl, N., Olsen, A., Sabine, C. & Heimann, M. 2013 Global surface-ocean p(CO_2) and sea-air CO_2 flux variability from an observation-driven ocean mixed-layer scheme. *Ocean Science* **9**, 193–216. (doi:10.5194/os-9-193-2013)

7. Van der Werf, G. R. et al. 2017 Global fire emissions estimates during 1997–2016. *Earth System Science Data* **9**, 697–720. (doi:10.5194/essd-9-697-2017)

8. Monteil, G. & Scholze, M. 2019 Regional CO_2 inversions with LUMIA, the Lund University Modular Inversion Algorithm, v1.0. *Geosci. Model Dev.* **2019**, 1–35. (doi:10.5194/gmd-2019-227)

9. Monteil, G. et al. 2019 The regional EUROpean atmospheric transport inversion COMparison, EUROCOM: first results on European wide terrestrial carbon fluxes for the period 2006-2015. *Atmos. Chem. Phys. Discuss.*, 1–38.

10. Rodenbeck, C., Gerbig, C., Trusilova, K. & Heimann, M. 2009 A two-step scheme for high-resolution regional atmospheric trace gas inversions based on independent models. *Atmos. Chem. Phys.* **9**, 5331–5342. (doi:10.5194/acp-9-5331-2009)

11. Basu, S. et al. 2013 Global CO_2 fluxes estimated from GOSAT retrievals of total column CO_2. *Atmos. Chem. Phys.* **13**, 8695–8717. (doi:10.5194/acp-13-8695-2013)

12. Rodenbeck, C., Houweling, S., Gloor, M. & Heimann, M. 2003 CO_2 flux history 1982-2001 inferred from atmospheric data using a global inversion of atmospheric transport. *Atmos. Chem. Phys.* **3**, 1919–1964.

13. Lin, J. C., Gerbig, C., Wofsy, S. C., Andrews, A. E., Daube, B. C., Davis, K. J. & Grainger, C. A. 2003 A near-field tool for simulating the upstream influence of atmospheric observations: The Stochastic Time-Inverted Lagrangian Transport (STILT) model. *J. Geophys. Res.* (doi:10.1029/2002JD003161)

14. Kountouris, P., Gerbig, C., Rödenbeck, C., Karstens, U., Koch, T. F. & Heimann, M. 2018 Atmospheric CO_2 inversions on the mesoscale using data-driven prior uncertainties: quantification of the European terrestrial CO_2 fluxes. *Atmos. Chem. Phys.* **18**, 3047–3064. (doi:10.5194/acp-18-3047-2018)

15. Fletcher, S. E. M. et al. 2007 Inverse estimates of the oceanic sources and sinks of natural CO_2 and the implied oceanic carbon transport. *Global Biogeochem. Cycles* **21**. (doi:10.1029/2006GB002751)

16. Manning, A. J., O'Doherty, S., Jones, A. R., Simmonds, P. G. & Derwent, R. G. 2011 Estimating UK methane and nitrous oxide emissions from 1990 to 2007 using an inversion modeling approach. *J. Geophys. Res.* **116**, D02305. (doi:10.1029/2010jd014763)

17. White, E. D. et al. 2019 Quantifying the UK's carbon dioxide flux: an atmospheric inverse modelling approach using a regional measurement network. *Atmos. Chem. Phys.* **19**, 4345–4365. (doi:10.5194/acp-19-4345-2019)

18. Takahashi, T. et al. 2009 Climatological mean and decadal change in surface ocean pCO_2, and net sea--air CO_2 flux over the global oceans. *Deep Sea Research Part II: Topical Studies in Oceanography* **56**, 554–577. (doi:10.1016/j.dsr2.2008.12.009)

19. Ganesan, A. L. et al. 2014 Characterization of uncertainties in atmospheric trace gas inversions using hierarchical Bayesian methods. *Atmos. Chem. Phys.* **14**, 3855–3864. (doi:10.5194/acp-14-3855-2014)

20. Lunt, M. F., Rigby, M., Ganesan, A. L. & Manning, A. J. 2016 Estimation of trace gas fluxes with objectively determined basis functions using reversible-jump Markov chain Monte Carlo. *Geosci. Model Dev.* **9**, 3213–3229. (doi:10.5194/gmd-9-3213-2016)

21. Burton, C., Betts, R., Cardoso, M., Feldpausch, T. R., Harper, A., Jones, C. D., Kelley, D. I., Robertson, E. & Wiltshire, A. 2019 Representation of fire, land-use change and vegetation dynamics in the Joint UK Land Environment Simulator vn4.9 (JULES). *Geosci. Model Dev.* **12**, 179–193. (doi:10.5194/gmd-12-179-2019)

22. Smith, B., Wårlind, D., Arneth, A., Hickler, T., Leadley, P., Siltberg, J. & Zaehle, S. 2014 Implications of incorporating N cycling and N limitations on primary production in an individual-based dynamic vegetation model. *Biogeosciences* **11**, 2027–2054. (doi:10.5194/bg-11-2027-2014)

23. Krinner, G., Viovy, N., de Noblet-Ducoudré, N., Ogée, J., Polcher, J., Friedlingstein, P., Ciais, P., Sitch, S. & Prentice, I. C. 2005 A dynamic global vegetation model for studies of the coupled atmosphere-biosphere system. *Global Biogeochem. Cycles* **19**. (doi:10.1029/2003GB002199)

24. Fortems-Cheiney, A., Pison, I., Dufour, G., Broquet, G., Berchet, A., Potier, E., Coman, A., Siour, G. & Costantino, L. 2019 Variational regional inverse modeling of reactive species emissions with PYVAR-CHIMERE. *Geosci. Model Dev.* **2019**, 1–22. (doi:10.5194/gmd-2019-186)

25. Menut, L. et al. 2013 CHIMERE 2013: a model for regional atmospheric composition modelling. *Geosci. Model Development* **6**, 981–1028. (doi:10.5194/gmd-6-981-2013)

26. Chevallier, F., Fisher, M., Peylin, P., Serrar, S., Bousquet, P., Breon, F. M., Chédin, A. & Ciais, P. 2005 Inferring CO_2 sources and sinks from satellite observations: Method and application to TOVS data. *J. Geophys. Res. Atmos.* **110**. (doi:10.1029/2005jd006390)

27. Broquet, G. et al. 2013 Regional inversion of CO_2 ecosystem fluxes from atmospheric measurements: reliability of the uncertainty estimates. *Atmos. Chem. Phys.* **13**, 9039–9056. (doi:10.5194/acp-13-9039-2013)

28. Broquet, G., Chevallier, F., Rayner, P., Aulagnier, C., Pison, I., Ramonet, M., Schmidt, M., Vermeulen, A. T. & Ciais, P. 2011 A European summertime CO_2 biogenic flux inversion at mesoscale from continuous in situ mixing ratio measurements. *J. Geophys. Res.* **116**, 1–22. (doi:10.1029/2011JD016202)

29. Kountouris, P. et al. 2015 An objective prior error quantification for regional atmospheric inverse applications. *Biogeosciences* **12**, 7403–7421. (doi:10.5194/bg-12-7403-2015)

Table 1. Atmospheric measurement sites included in the inversions

Site code	Site name	Institute	Latitude	Longitude	Altitude (masl)	Sample Height (magl)
BIK*	Bialystok, Poland	MPI-BGC	53.23	23.03	183	300
BIR*	Birkenes, Norway	NILU	58.39	8.25	219	2.5
BIS*	Biscarosse, France	LSCE	44.38	-1.23	73	47
BRM	Beromunster, Switzerland	KUP	47.19	8.18	797	212
BSD	Bilsdale, UK	UNIVBRIS	54.36	-1.15	380	248
CBW*	Cabauw, The Netherlands	TNO	51.97	4.93	-1	207
CIB	Centro de Investigacion de la Baja Atmosfera, Spain	CIBA	41.81	-4.93	845	5
CMN*	Monte Cimone, Italy	CNR-ISAC	44.17	10.68	2165	12
CRP	Carnsore Point, Ireland	EPA	52.18	-6.37	9	14
DEC	Delta de l'Ebre, Spain	ICTA-UAB	40.74	0.79	1	10
EEC	El Estrecho, Spain	ICTA-UAB	36.06	-5.66	20	20
ERS	Ersa, France	LSCE	42.97	9.38	533	40
FKL	Finokalia, Greece	ECPL	35.34	25.67	150	15
GAT	Gatrow, Germany	DWD	53.07	11.44	70	341
GIC	Sierra de Gredos, Spain	ICTA-UAB	40.35	-5.18	1436	20
HEI*	Heidelberg, Germany	UHEI-IUP	49.42	8.67	116	30
HPB	Hohenpeissenberg, Germany	DWD	47.8	11.02	934	131
HTM	Hyltemossa, Sweden	CEC	56.1	13.42	115	150
HUN*	Hegyhatsal, Hungary	HMS	46.95	16.65	248	115
IPR	Ispra, Italy	JRC	45.81	8.64	210	100
IZO*	Izana, Tenerife, Canary Islands	AEMET	28.31	-16.5	2373	13
JFJ*	Jungfraujoch, Switzerland	EMPA	46.55	7.99	3570	10
KAS*	Kasprowy Wierch, Poland	AGH	49.23	19.98	1989	5
KRE	Kresin u Pacova, Czech Republic	CAS	49.57	15.08	534	250
LHW	Laegern-Hochwacht, Switzerland	EMPA	47.48	8.4	840	32
LIN	Lindenberg, Germany	DWD	52.17	14.12	73	98
LMP	Lampedusa, Italy	ENEA	35.53	12.52	45	10
LMU	La Muela, Spain	ICTA-UAB	41.59	-1.1	571	80
LUT*	Lutjewad, The Netherlands	CIO-RUG	53.4	6.35	1	60
MHD*	Mace Head, Ireland	NUI	53.33	-9.9	5	24
MLH	Malin Head, Ireland	EPA	55.35	-7.33	22	47
NOR	Norunda, Sweden	CEC	60.09	17.48	46	100
OHP	Observatoire de Haute Provence, France	OSU	43.93	5.71	650	100
OPE	Observatorie Pérenne de l'Environnement, France	ANDRA	48.56	5.5	390	120
PAL*	Pallas-Sammaltunturi, Finland	FMI	67.97	24.12	565	5
PDM	Pic du Midi, France	LSCE	42.94	0.14	2877	10
PRS*	Plateau Rosa, Italy	RSE	45.93	7.7	3480	10

PUI	Puijo, Finland	UEF	62.91	27.65	232	79
PUY	Puy de Dome, France	LSCE	45.77	2.97	1465	10
RGL	Ridge Hill, UK	UNIVBRIS	52	-2.54	204	90
SAC	Saclay, France	CEA	48.72	2.14	160	100
SMR	Hyytiälä, Finland	INAR	61.85	24.29	181	125
SSL*	Schauinsland, Germany	UBA	47.92	7.92	1205	12
SVB	Svartberget, Sweden	SLU	64.26	19.77	235	150
TAC	Tacolneston, UK	UNIVBRIS	52.52	1.14	56	185
TRN	Trainou, France	LSCE	47.96	2.11	131	180
TTA	Tall Tower Angus, UK	UNIVBRIS	56.55	-2.99	400	222
UTO	Utö, Finland	FMI	59.78	21.37	8	57
VAC	Valderejo, Spain	ICTA-UAB	42.88	-3.21	1102	20
WAO*	Weybourne, UK	UEA	52.95	1.12	20	10
WES	Westerland, Germany	UBA	54.93	8.32	12	0
ZEP	Ny-Alesund, Norway	NILU	78.91	11.89	474	15

*Sites having quasi-continuous observations for 2009-2018 used in inversions labelled "select".

Table 2. Atmospheric measurement sites used for the validation

Site code	Site name	Institute	Latitude	Longitude	Altitude (masl)	Sample Height (magl)
BAL	Baltic Sea, Poland	NOAA	55.50	16.67	3	25
BSC	Black Sea, Romania	NOAA	44.18	28.66	0	5
CIB	CIBA, Spain	NOAA	41.81	-4.93	845	5
HPB	Hohenpeissenberg, Germany	NOAA	47.8	11.02	985	5
OXK	Ochsenkopf, Germany	NOAA	50.03	11.81	1022	163
PDM	Pic du Midi, France	LSCE	42.94	0.14	2877	28

Table 3. Comparison of prior and posterior calculated CO_2 mole fractions at validation sites.

Model	RMSE		R^2		NSD*	
	Prior	Posterior	Prior	Posterior	Prior	Posterior
CarboScope Regional	8.32	8.18	0.59	0.62	0.90	0.99
LUMIA	46.05	46.57	0.30	0.26	0.65	0.66
PyVAR-CHIMERE	9.03	7.60	0.39	0.63	0.56	0.84
FLEXINVERT	5.87	5.89	0.67	0.68	1.01	1.02
NAME-HB	9.63	8.97	0.51	0.55	0.88	0.84

*NSD is Normalized Standard Deviation

Table 4. Eddy covariance flux sites from the Fluxnet network

Site code	Site name	IGBP code*	Latitude	Longitude	Altitude
BE-Bra	Brasschaat	MF	51.31	4.52	16
BE-Lon	Lonzee	CRO	50.55	4.75	167
CH-Cha	Chamau	GRA	47.21	8.41	393
CH-Dav	Davos	ENF	46.82	9.86	1639
CH-Fru	Früebüel	GRA	47.12	8.54	982
CH-Lae	Laegern	MF	47.48	8.36	689
CH-Oe2	Oensingen	CRO	47.29	7.73	452
CZ-BK1	Bily Kriz	ENF	49.50	18.54	875
CZ-wet	Trebon	WET	49.02	14.77	426
DE-Geb	Gebesee	CRO	51.10	10.91	162
DE-Gri	Grillenburg	GRA	50.95	13.51	385
DE-Hai	Hainich	DBF	51.08	10.45	430
DE-Kli	Klingenberg	CRO	50.89	13.52	478
DE-Obe	Oberbärenburg	ENF	50.79	13.72	734
DE-Tha	Tharandt	ENF	50.96	13.57	385
DK-Sor	Soroe	DBF	55.49	11.64	40
FI-Hyy	Hyytiala	ENF	61.85	24.29	181
IT-BCi	Borgo Cioffi	CRO	40.52	14.96	20
IT-Tor	Torgnon	GRA	45.84	7.58	2160
NL-Loo	Loobos	ENF	52.17	5.74	25
SE-Deg	Degero	WET	64.18	19.56	unknown

*MF = mixed forest, CRO = cropland, GRA = grasslands, ENF = evergreen needle leaf, DBF = deciduous broadleaf, WET = permanent wetlands

Figure 1. Taylor diagrams of the comparison of prior and posterior modelled mole fractions to observations (using all individual data points) for sites common to all inversions (CMN, HEI, HUN, JFJ, KAS, LUT, MHD, PAL, SSL, WAO) for the example year 2015. Note the plots have different axis extents.

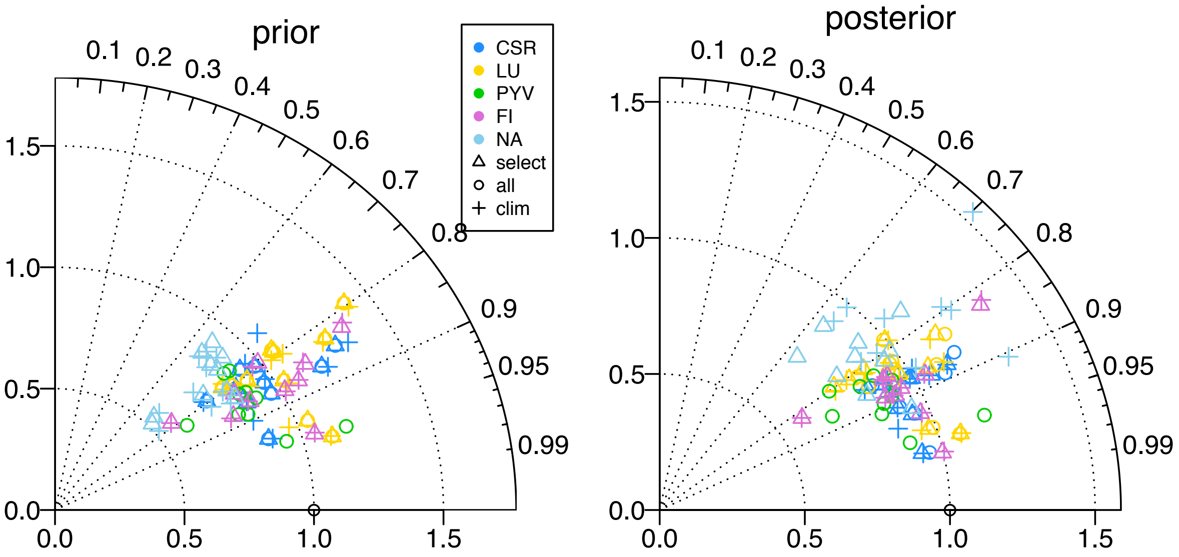

Figure 2. Histograms of the prior (blue) and posterior (red) model-observation errors for sites common to all inversions (CMN, HEI, HUN, JFJ, KAS, LUT, MHD, PAL, SSL, WAO) shown for the example year 2015. The y-axis is the probability density.

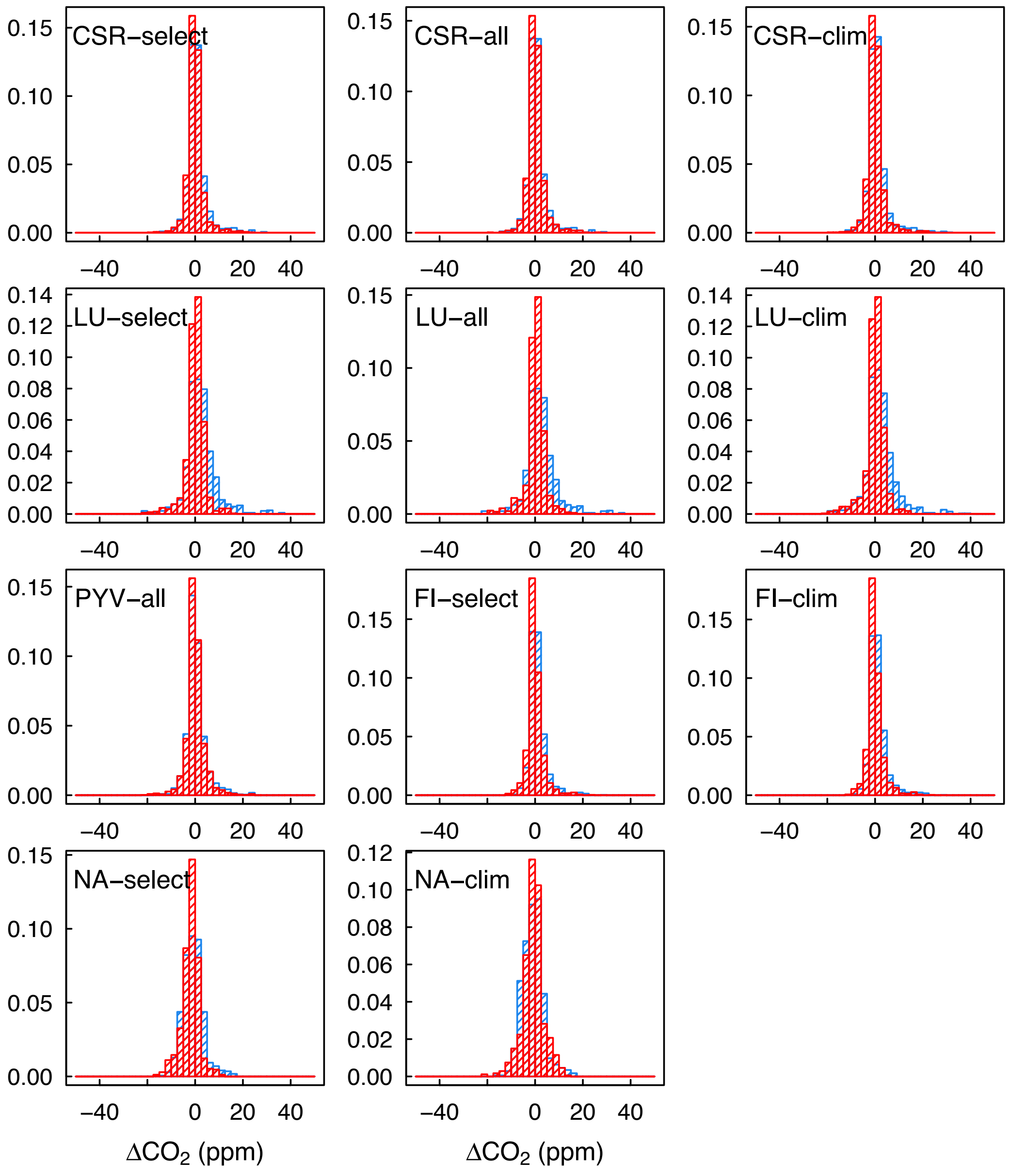

Figure 3. Taylor diagrams of the comparison of prior and posterior modelled mole fractions to validation sites (BAL, BSC, CIB, HPB, OXK, PDM) for the example year 2011. Note the plots have different axis extents.

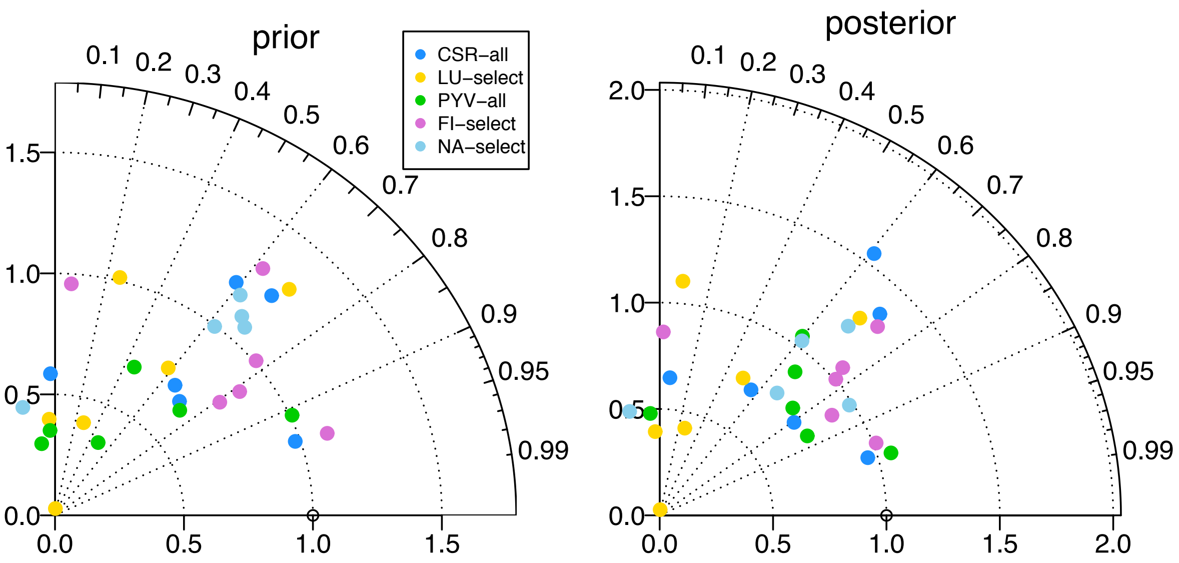

Figure 4. Annual NEE values (PgC y^{-1}) for the sum of the three regions for 2009-2018 shown for the priors (top) and the inversions (bottom). Also given are the means and standard deviations over all years for each prior/inversion. The colour scale is annual NEE (PgC y^{-1}).

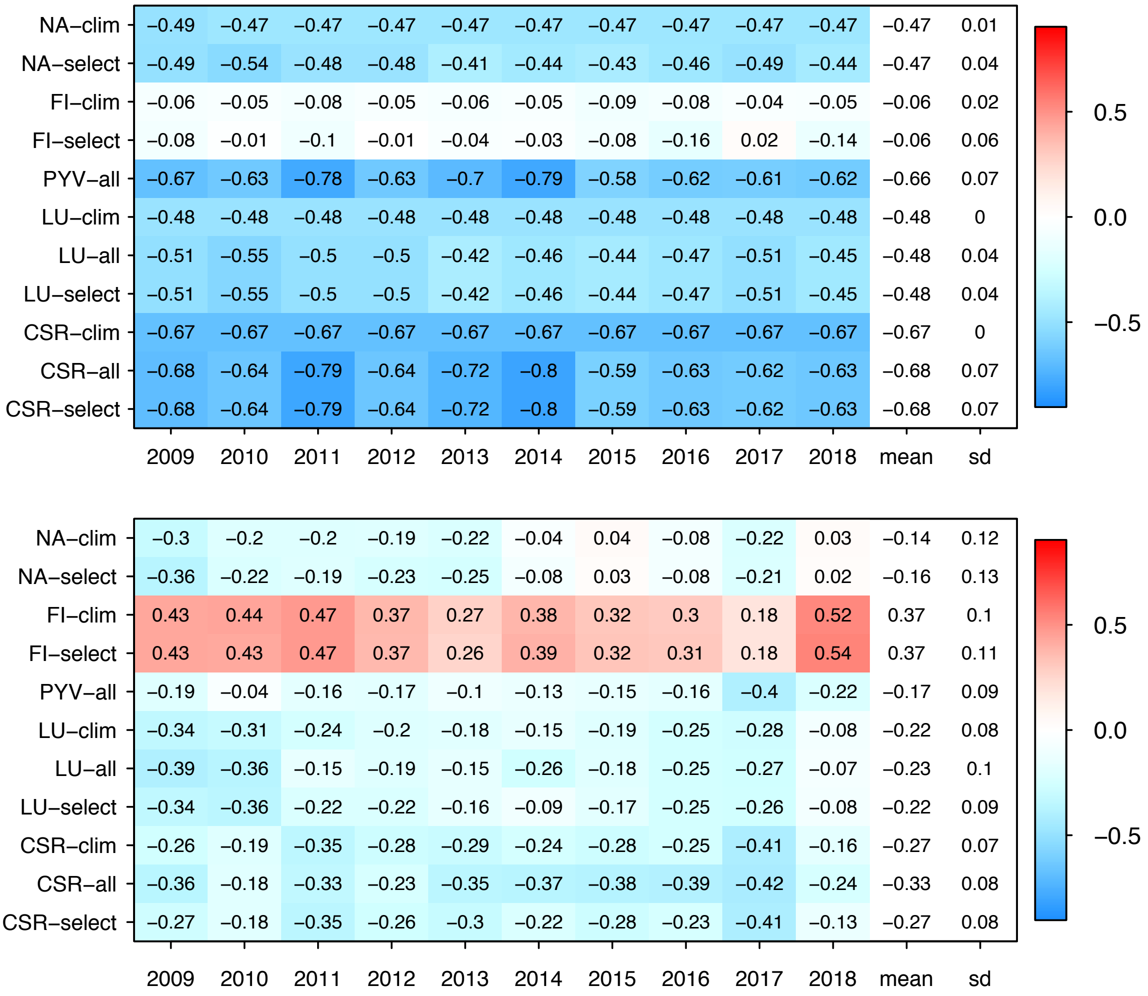

Figure 5. Meteorological and NDVI anomalies for 2018 relative to the 2009-2018 mean using ECMWF ERA5 reanalysis data and MODIS satellite data, respectively, a-d) 2m temperature for spring (March-May), summer (June-August), autumn (September-November) and for the duration of the drought (May-November), respectively; e-h) downward shortwave radiation; i-l) total precipitation; m-p) soil water volume for 0-7 cm depth and q-t) NDVI.

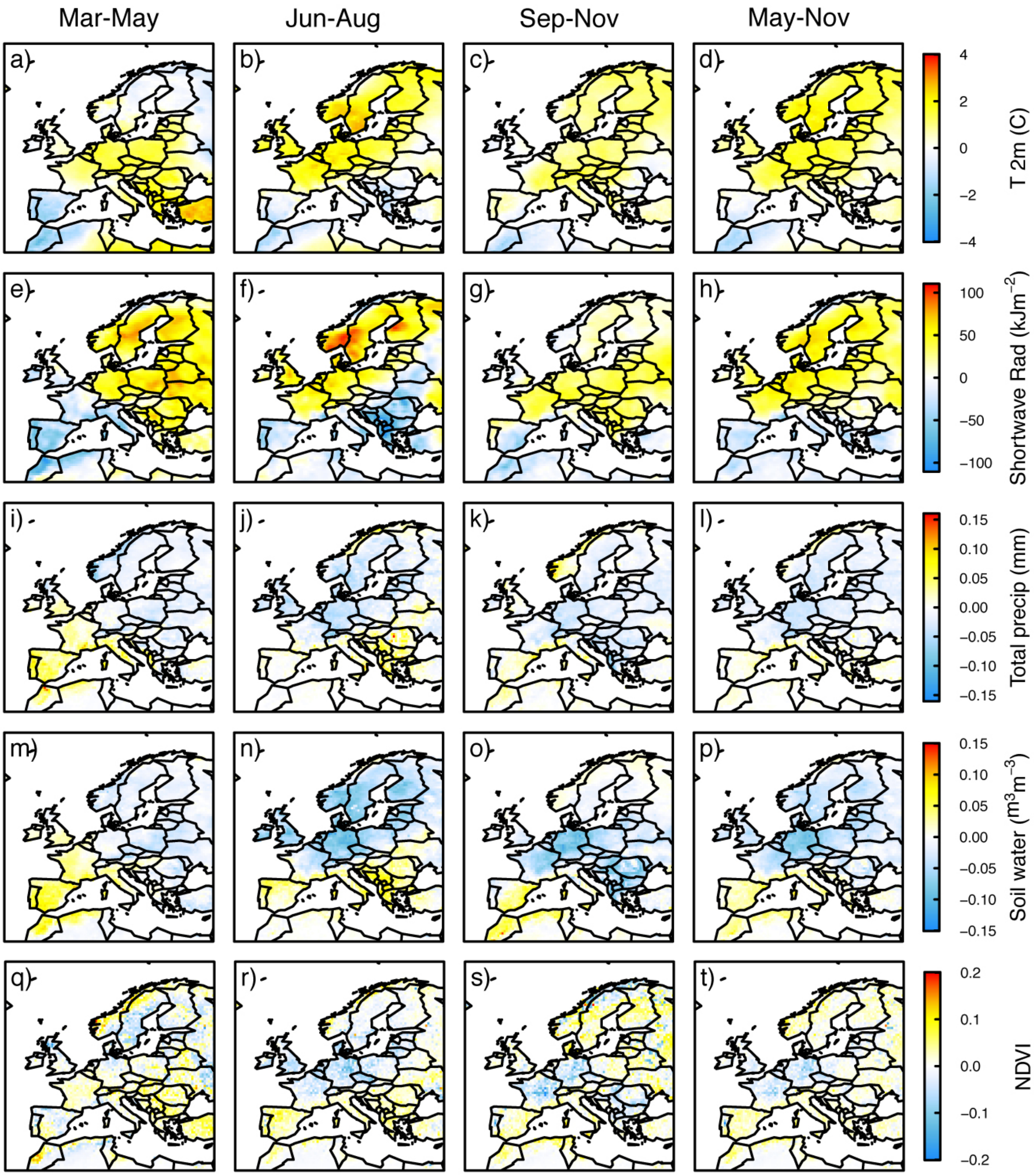

Figure 6. Annual mean NEE anomaly for the North, Temperate and Mediterranean regions shown for all inversions.

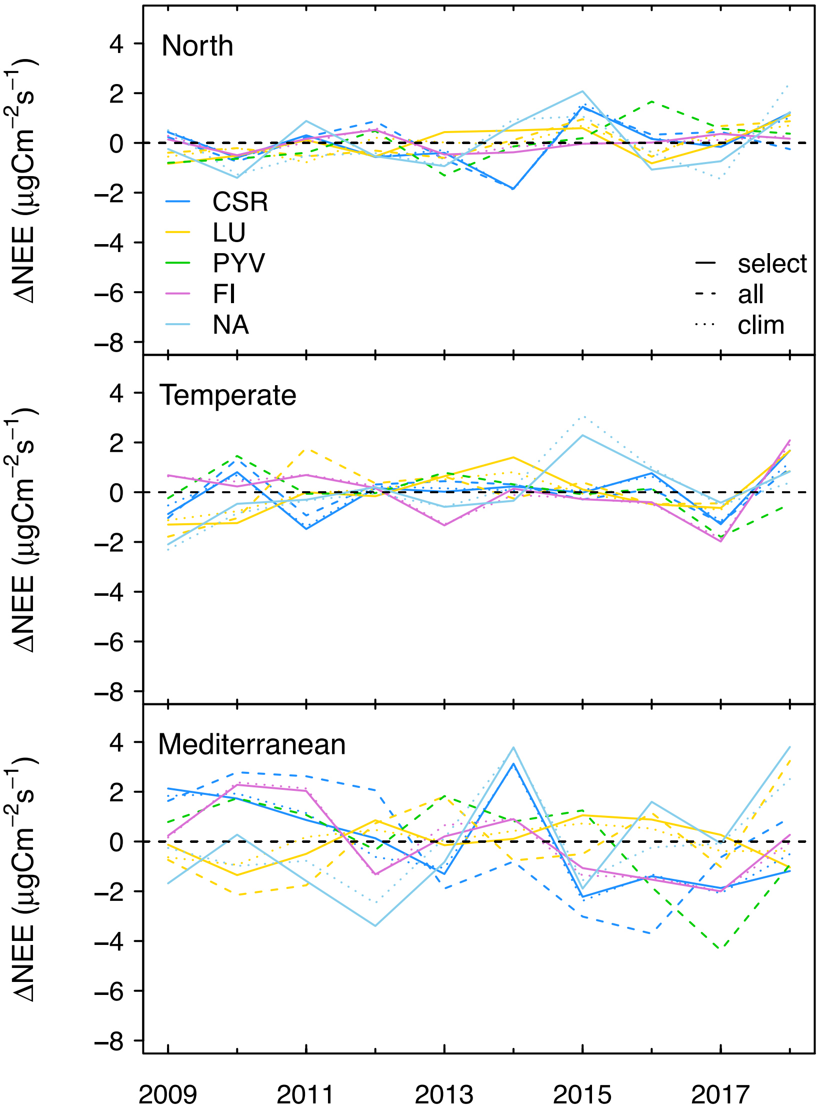

Figure 7. Monthly mean NEE anomaly for the North, Temperate and Mediterranean regions shown for all inversions.

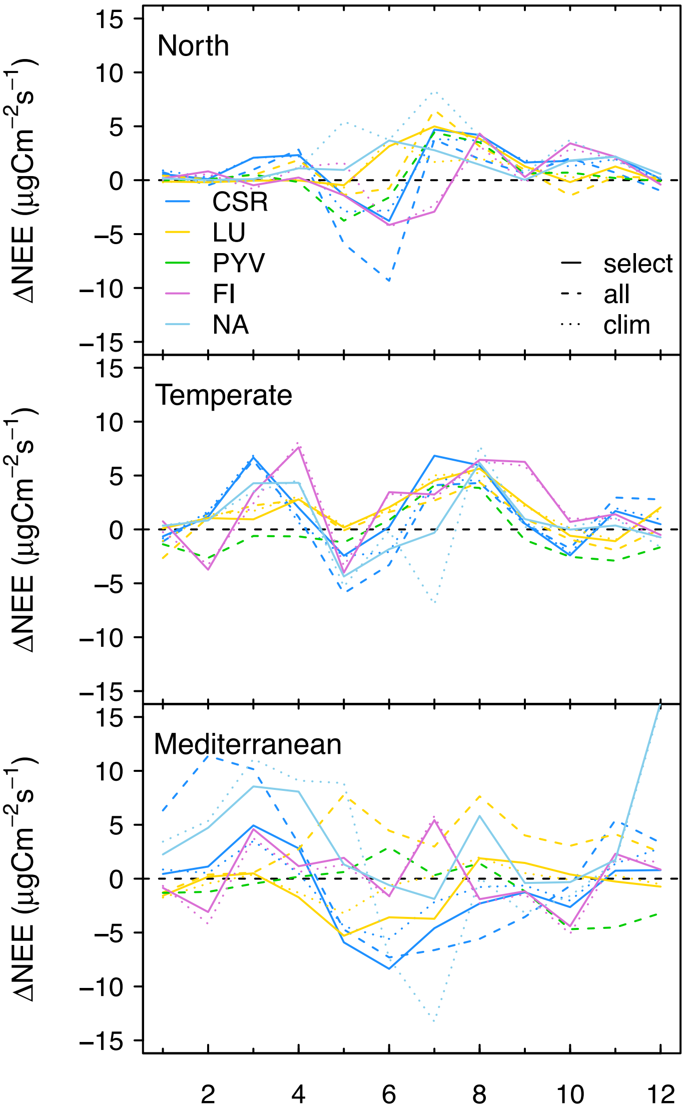

Figure 8. Map of eddy covariance flux sites. The regions used in the analysis are indicated by the colour shading.

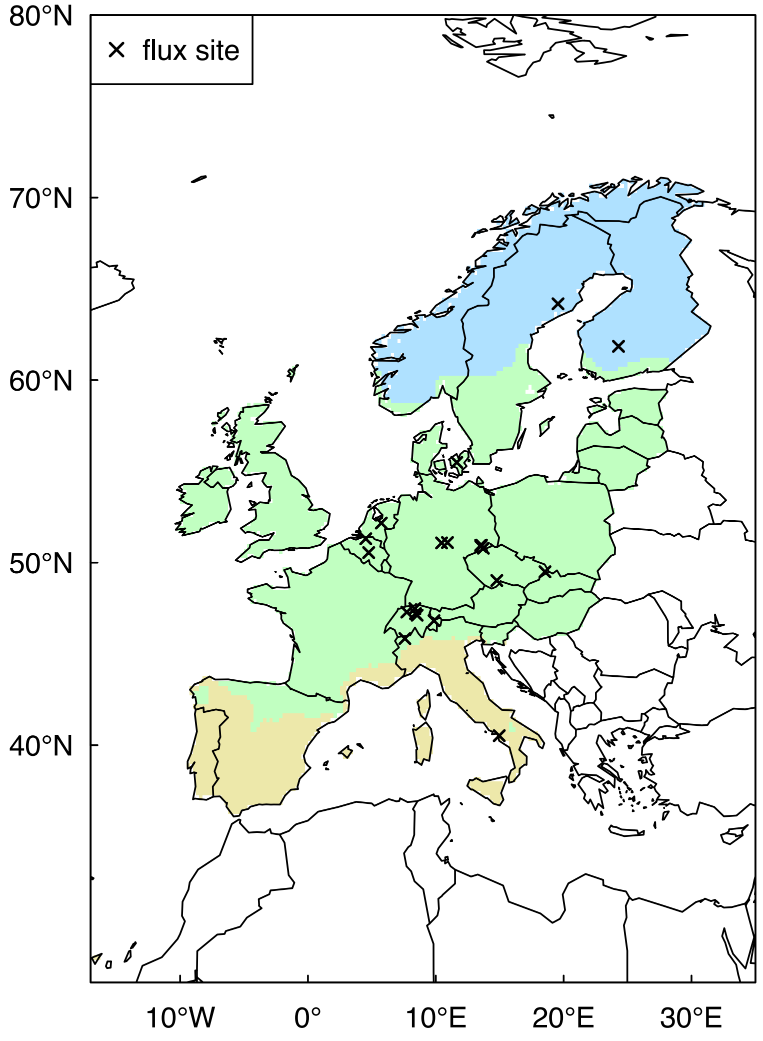

Figure 9. Spring (left), summer (centre) and annual (right) NEE anomalies (μgC m^{-2} s^{-1}) for 2018 compared to the mean 2009-2018 for each inversion.

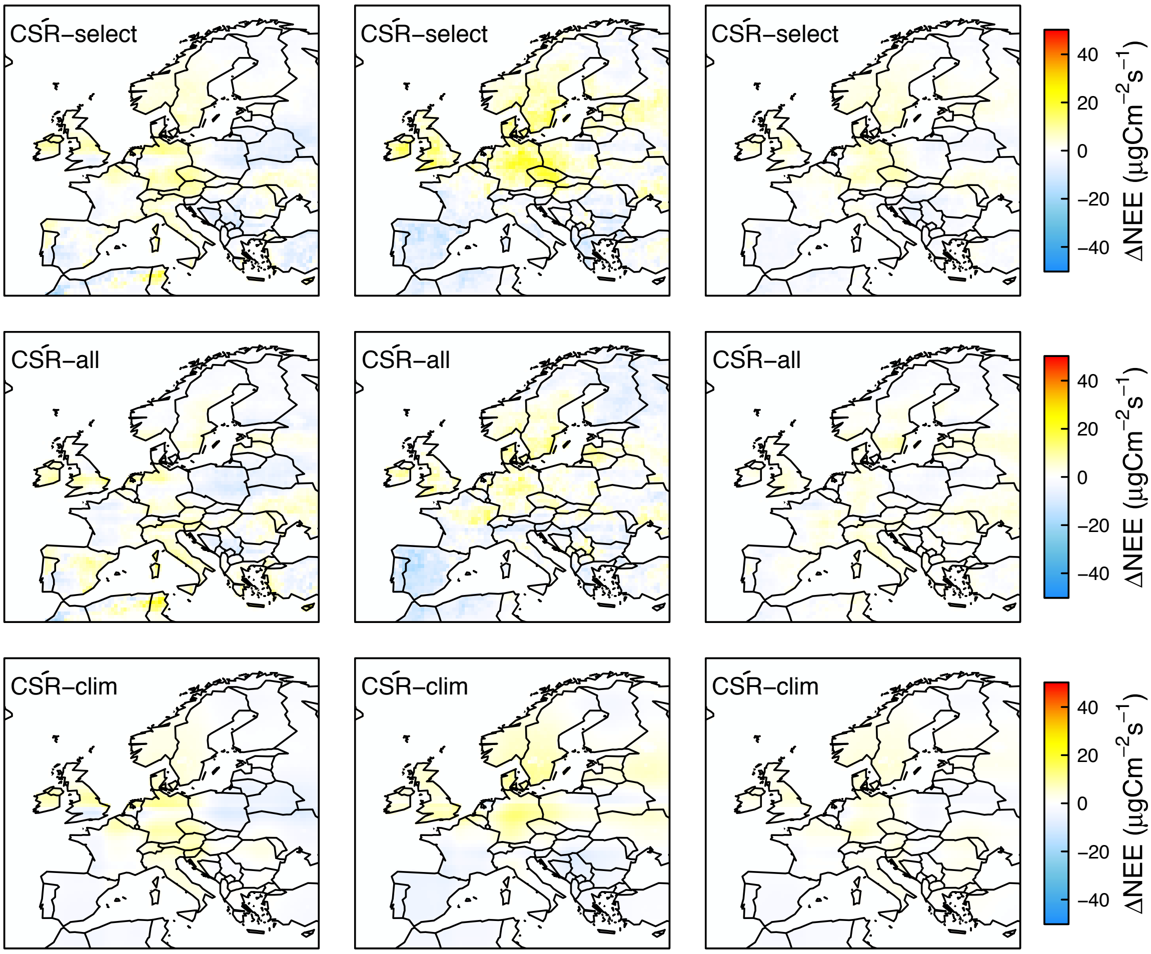

Figure 9. continued

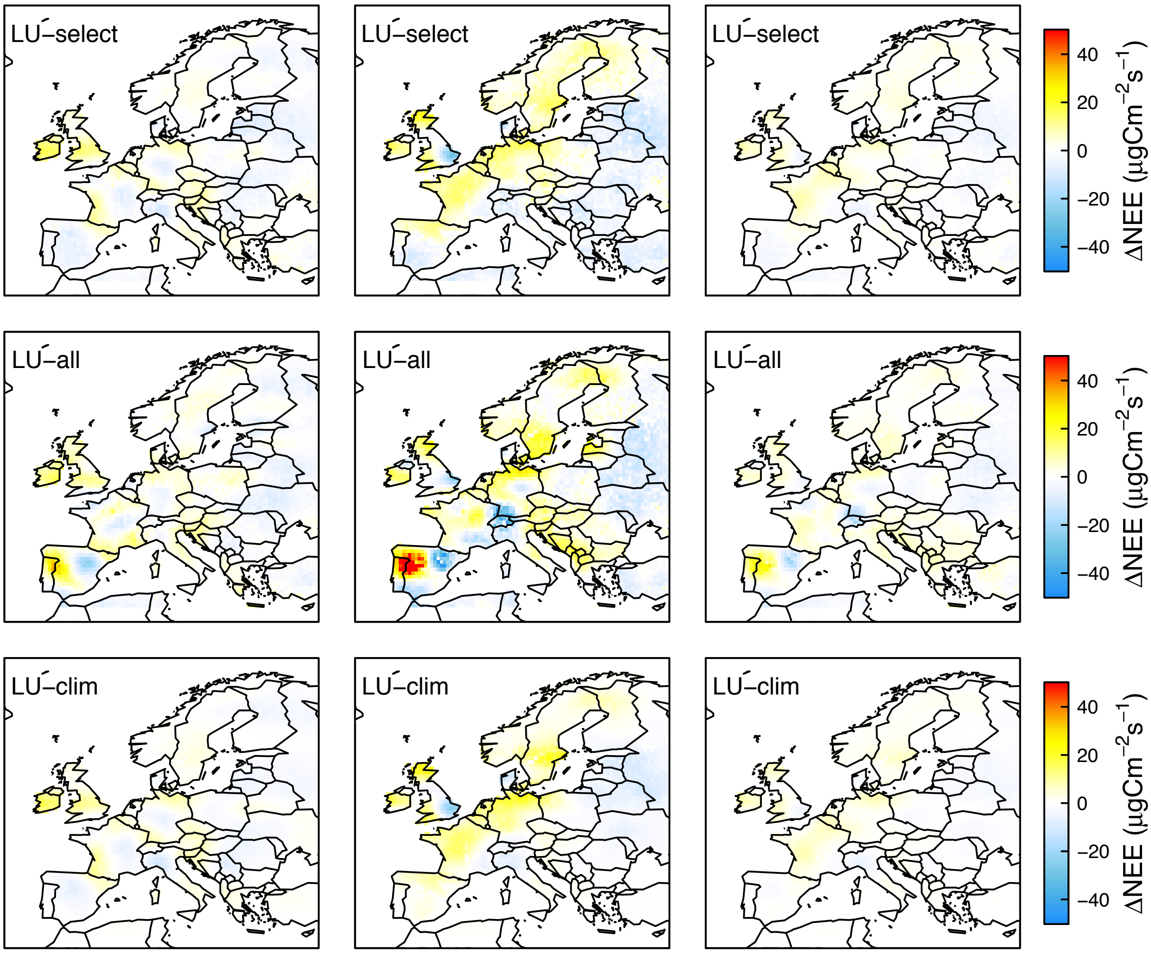

Figure 9. continued

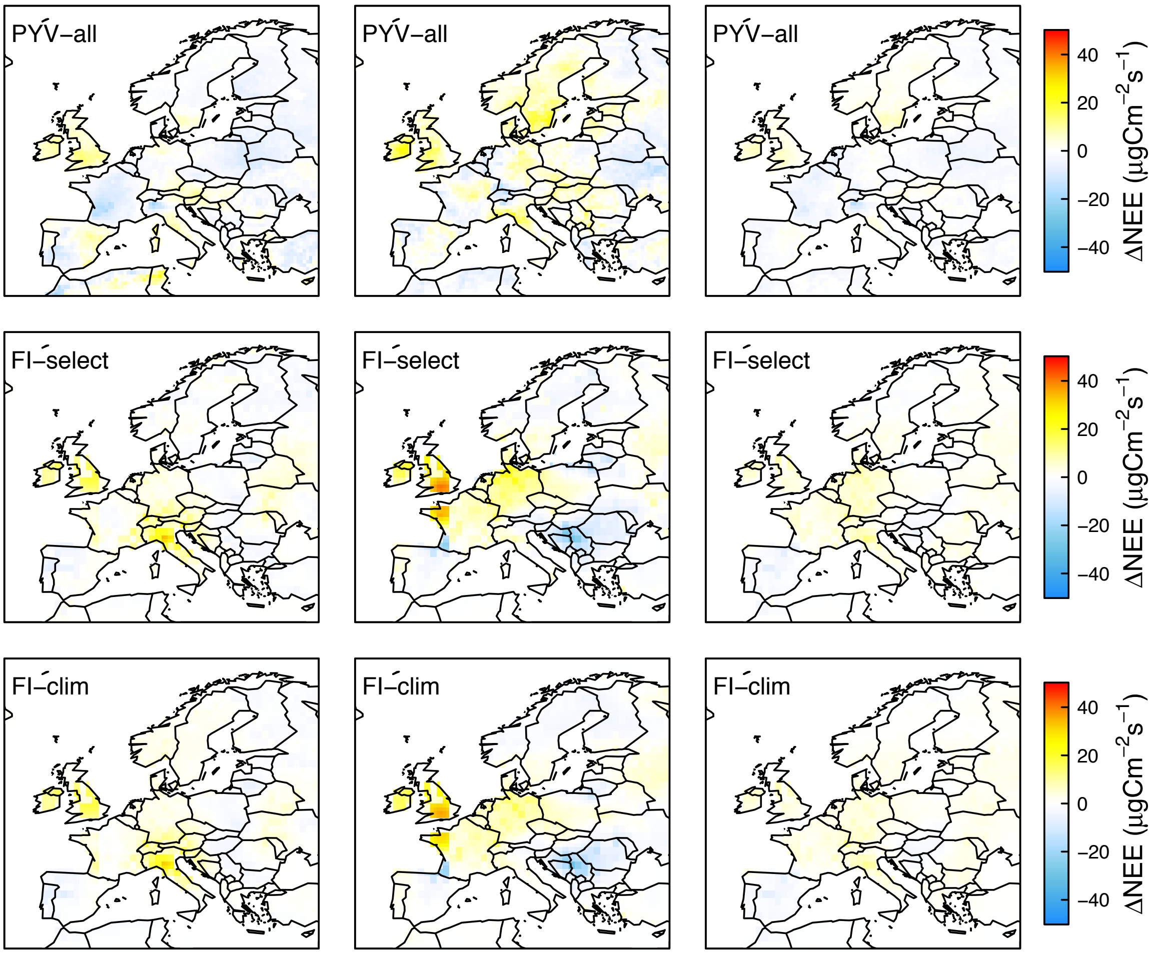

Figure 9. continued

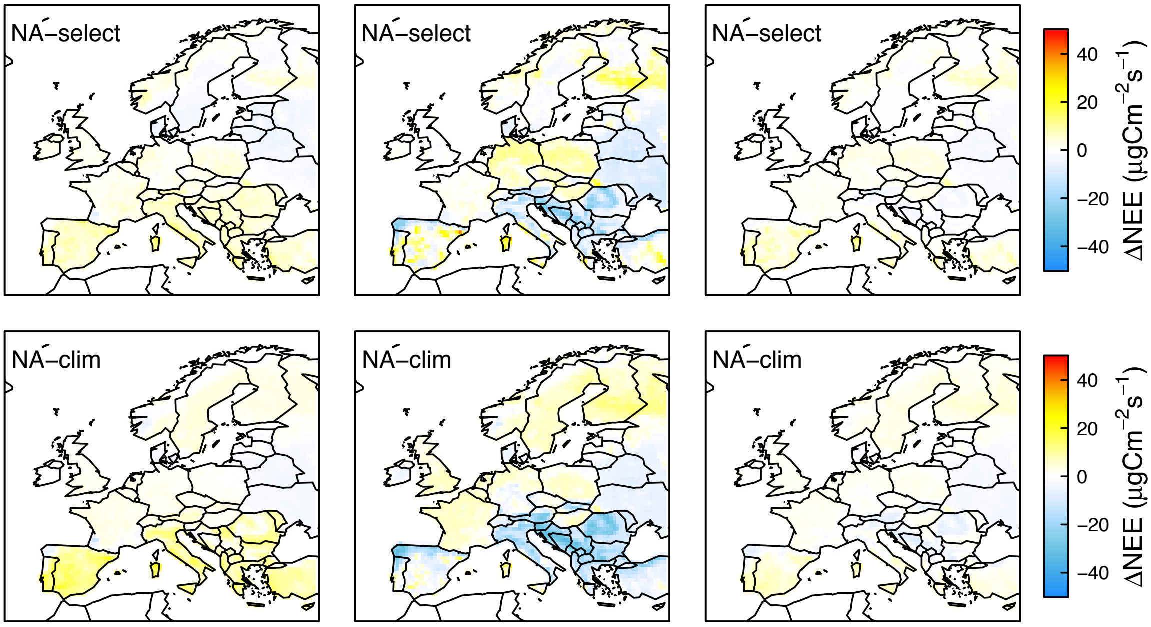

Supplement: Supplementary Information for ‘Changes in net ecosystem exchange over Europe during the 2018 drought based on atmospheric observations’ [file rstb20190512supp1.pdf]
